# Prefoldin Promotes Proteasomal Degradation of Cytosolic Proteins with Missense Mutations by Maintaining Substrate Solubility

**DOI:** 10.1371/journal.pgen.1006184

**Published:** 2016-07-22

**Authors:** Sophie A. Comyn, Barry P. Young, Christopher J. Loewen, Thibault Mayor

**Affiliations:** 1 Michael Smith Laboratories, University of British Columbia, Vancouver, British Columbia, Canada; 2 Genome Science and Technology Program, University of British Columbia, Vancouver, British Columbia, Canada; 3 Department of Cellular and Physiological Sciences, University of British Columbia, Vancouver, British Columbia, Canada; 4 Department of Biochemistry and Molecular Biology, University of British Columbia, Vancouver, British Columbia, Canada; University of Washington, UNITED STATES

## Abstract

Misfolded proteins challenge the ability of cells to maintain protein homeostasis and can accumulate into toxic protein aggregates. As a consequence, cells have adopted a number of protein quality control pathways to prevent protein aggregation, promote protein folding, and target terminally misfolded proteins for degradation. In this study, we employed a thermosensitive allele of the yeast Guk1 guanylate kinase as a model misfolded protein to investigate degradative protein quality control pathways. We performed a flow cytometry based screen to identify factors that promote proteasomal degradation of proteins misfolded as the result of missense mutations. In addition to the E3 ubiquitin ligase Ubr1, we identified the prefoldin chaperone subunit Gim3 as an important quality control factor. Whereas the absence of *GIM3* did not impair proteasomal function or the ubiquitination of the model substrate, it led to the accumulation of the poorly soluble model substrate in cellular inclusions that was accompanied by delayed degradation. We found that Gim3 interacted with the Guk1 mutant allele and propose that prefoldin promotes the degradation of the unstable model substrate by maintaining the solubility of the misfolded protein. We also demonstrated that in addition to the Guk1 mutant, prefoldin can stabilize other misfolded cytosolic proteins containing missense mutations.

## Author Summary

Most polypeptides by necessity must fold into three-dimensional structures in order to become functional proteins. Misfolding, either during or subsequent to initial folding, can result in toxic protein aggregation. As a consequence, cells have adopted a number of protein quality control pathways to prevent protein aggregation, promote protein folding, and target terminally misfolded proteins for degradation. One cause of misfolding is the presence of missense mutations, which account for over half of all the reported mutations in the Human Gene Mutation Database. Here we establish a model cytosolic protein substrate whose stability is temperature dependent. We then perform a flow cytometry based screen to identify factors that promote the degradation of our model substrate. We identified the E3 ubiquitin ligase Ubr1 and the prefoldin chaperone complex subunit Gim3. Prefoldin forms a “jellyfish-like” structure and aids in nascent protein folding and prevents protein aggregation. We show that prefoldin promotes protein degradation by maintaining substrate solubility. Our work adds to that of others highlighting the importance of the prefoldin complex in preventing potentially toxic protein aggregation.

## Introduction

The protein homeostasis network encompasses systems required by the cell to generate and maintain the correct levels, conformational state, and distribution of its proteome [1]. Misfolded proteins threaten this balance by triggering loss of function phenotypes, diverting resources away from producing essential protein products, or precipitating the production of potentially toxic protein aggregates [2]. The presence of protein aggregates is characteristic of a number of neurodegenerative diseases such as Parkinson’s and Alzheimer’s disease, and a decrease in the protein homeostasis capacity of the cell is thought to underlie the later stages of cellular ageing [3–5]. It is, therefore, not surprising that the cell has evolved a number of protein quality control pathways aimed at preventing protein aggregation, promoting protein folding, and targeting terminally misfolded proteins for degradation [6–8]. These pathways triage misfolded proteins, which will face three main possible fates: to be refolded back to their functional native conformation; to be targeted for degradation; or to be sequestered into spatially distinct quality control compartments.

Proteins are selectively targeted to the eukaryotic ubiquitin proteasome system by the covalent attachment of polyubiquitin chains catalyzed by a cascade of E1 (ubiquitin-activating), E2 (ubiquitin-conjugating), and E3 (ubiquitin ligase) enzymes [9, 10]. Substrate recruitment and specificity is determined by the E3 ubiquitin ligases, either alone or in concert with an E2 conjugating enzyme or other substrate adaptors. A number of subcellular compartment-specific quality control pathways have been identified, each associated with a particular E3 ligase or set of ligases [6, 11, 12]. In yeast, the San1 ligase is responsible for ubiquitinating nuclear misfolded proteins [12]. Experiments have shown that San1 binds misfolded proteins through recognition sequences located in disordered regions of its N- and C-terminal domains [13]. In contrast to the nucleus, a number of ligases have been identified to target cytosolic proteins for degradation in yeast. While initially characterized for its role as the recognin of the N-end rule pathway, Ubr1 has also been shown to target misfolded cytoplasmic proteins for degradation [14–18]. It does so either alone, or in conjunction with other E3 ligases such as Ubr2 in the case of newly synthesized kinases, or with the nuclear San1 where both are required for the complete degradation of the engineered ∆ssCPY*-GFP substrate [15, 16]. Hul5, a nuclear protein that relocalizes to the cytoplasm upon heat shock, and Rsp5 have been identified as the two ligases responsible for the marked increase in cytoplasmic protein ubiquitination following heat shock stress [19, 20]. Finally, the ribosome associated ligase Ltn1 targets non-stop polypeptides stalled during translation for degradation [21].

Recently, the importance of spatial organization in protein quality control has gained recognition. Under normal physiological conditions, misfolded proteins can be concentrated into dynamic Q-bodies where they can be refolded by chaperones or degraded [22]. However, if the protein quality control systems become overwhelmed, misfolded proteins can be sequestered into discrete cellular inclusions. The juxtanuclear quality control compartment (JUNQ) acts to concentrate detergent soluble misfolded proteins capable of being refolded, or degraded, and contains 26S proteasomes and chaperones such as the disaggregase Hsp104 [23, 24]. The insoluble protein deposit (IPOD) by contrast contains insoluble non-ubiquitinated proteins; does not co-localize with proteasomes; and is the site of amyloidogenic protein sequestration, perhaps to prevent their toxic interaction with quality control machinery [23]. The IPOD is also postulated to be the site of yeast prion maturation [25].

In this study we performed a screen to identify factors involved in degradative protein quality control of a model substrate that misfolds as the result of destabilizing missense mutations. We show that our model substrate is thermally unstable, undergoes proteasome mediated degradation, and forms Q-body like inclusions. We then identified and characterized the prefoldin chaperone subunit Gim3 as a factor important for maintaining our substrate protein’s solubility, and thereby facilitating its degradation.

## Results

### Guk1-7 is thermally unstable

**Fig 1:**
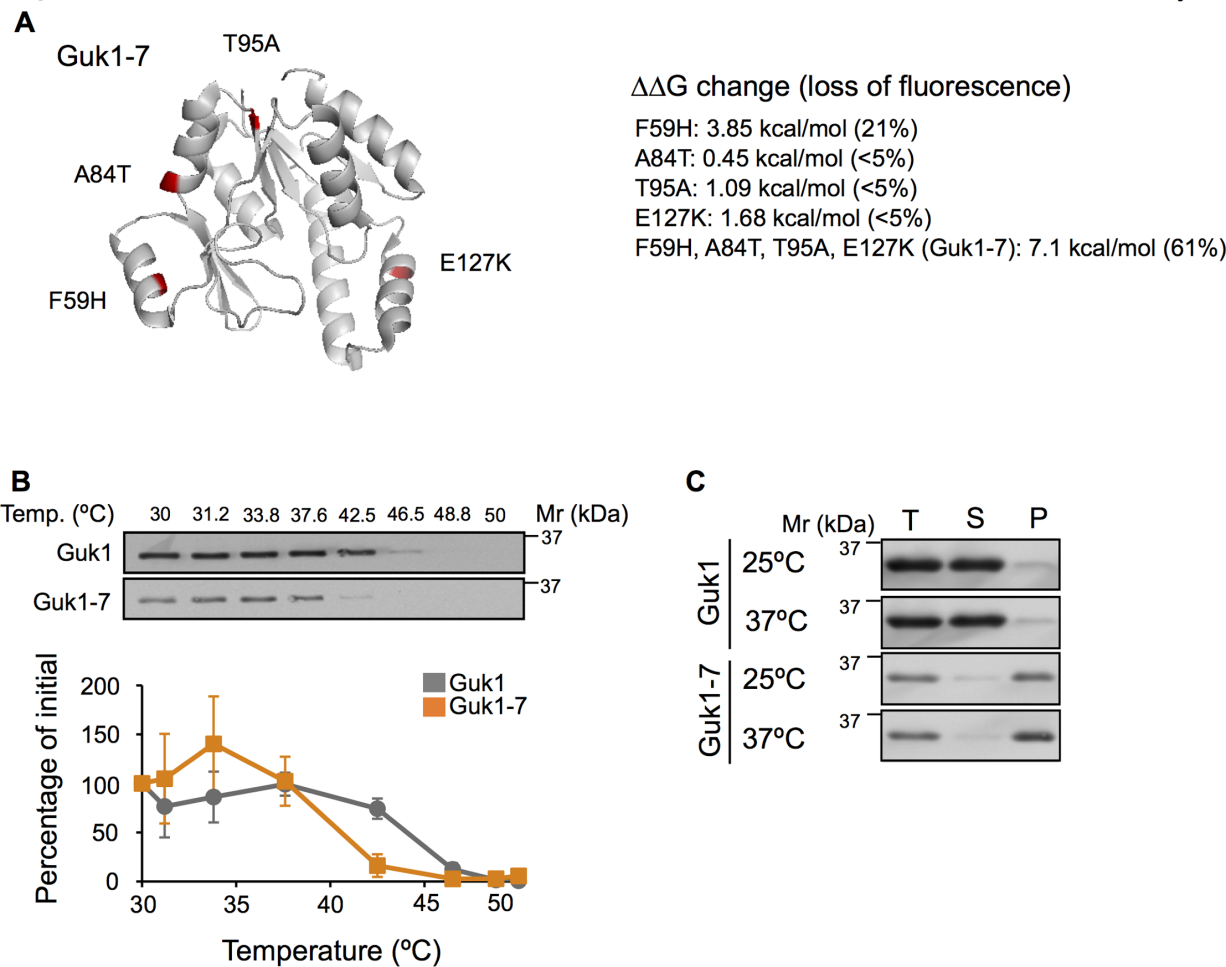
Guk1-7 is thermally unstable. (A) Ribbon structure of Guk1 (PDB 1EX7). Positions of the four missense mutations and predicted ∆∆G values are indicated. Loss of fluorescence measured by flow cytometry after a two hour incubation at 37°C with cycloheximide is indicated in brackets. (B) Cellular thermal shift assay of Guk1 and Guk1-7 fused to a six histidine tag in lysates derived from cells grown at 25°C. One representative anti-His Western Blot is shown. The graph represents the means and standard deviations of Guk1 levels from three independent experiments. (C) Guk1 and Guk1-7 fused to a six histidine tag were expressed in cells grown at 25°C or shifted to 37°C for 20 min. Total cell lysate (T), soluble (S), and pellet fractions (P) were immunoblotted with anti-His antibodies.

Our lab previously identified a panel of temperature sensitive alleles of essential genes encoding for cytosolic proteins in *Saccharomyces cerevisiae* [26]. A large fraction of mutant proteins underwent proteasome-mediated degradation when incubated at the restrictive temperature of 37°C, whereas the wild type proteins were stable. While approximately one third of the unstable alleles were found to be substrates of the E3 ubiquitin ligase Ubr1 [26], the protein quality control pathways responsible for the proteasomal degradation of the remaining mutant proteins are unknown. To screen for other proteins involved in proteasome mediated degradation of thermosensitive mutant proteins, we sought to establish an assay based on fluorescence intensity to facilitate the quantification of a model quality control substrate fused to GFP. For this study, we selected the Guk1-7 allele that contains four missense mutations generated by random PCR-based mutagenesis [26–28]. Guk1 is a member of the nucleoside monophosphate kinase (NMP) family and converts GMP to GDP [29]. Similar to other members of the NMP family, Guk1’s structure contains a core, a lid, and a dynamic NMP-binding domain [30]. Mutants of Guk1 are defective in mannose chain elongation, have higher cell wall porosity, and are hypersensitive to larger molecular weight antibiotics [31]. We first predicted the structural stability effects of the missense mutations found in Guk1-7 using FoldX (Fig 1A) [32]. The predicted free energy changes (∆∆G) between the single point mutants and the wild type protein were modest, whereas the combined effect of all the mutations found in Guk1-7 was much larger (~7 kcal/mol). While this value is higher than that predicted for missense mutations in transmembrane domains of disease-associated proteins such as cystic fibrosis transmembrane conductance regulator (CFTR) and rhodopsin (1.5 and 1.9 kcal/mol, respectively), it is in line with those predicted for mutations in phenylalanine hydroxylase (PAH) associated with mild or severe forms of phenylketonuria (5.7 and 14.2 kcal/mol, respectively) [33, 34]. We then compared the thermodynamic stability of ectopically expressed wild type Guk1 with Guk1-7 in cellular lysates by a cellular thermal shift assay (CETSA) [35]. In agreement with its predicted lower stability, Guk1-7 was less stable than the wild type Guk1 at incubation temperatures above 38°C (Fig 1B). We further examined the solubility of Guk1 and Guk1-7 proteins in cells incubated at the normal growth temperature of 25°C and following a short 20 minute incubation at 37°C. Guk1 was found predominantly in the soluble form while Guk1-7 was enriched in the NP-40 insoluble fraction at 25°C and 37°C (Fig 1C). Together these data suggest that Guk1-7 is much less stable than the wild type protein and misfolds forming NP-40 insoluble aggregates.

### Fluorescence-based assay to assess protein stability

**Fig 2:**
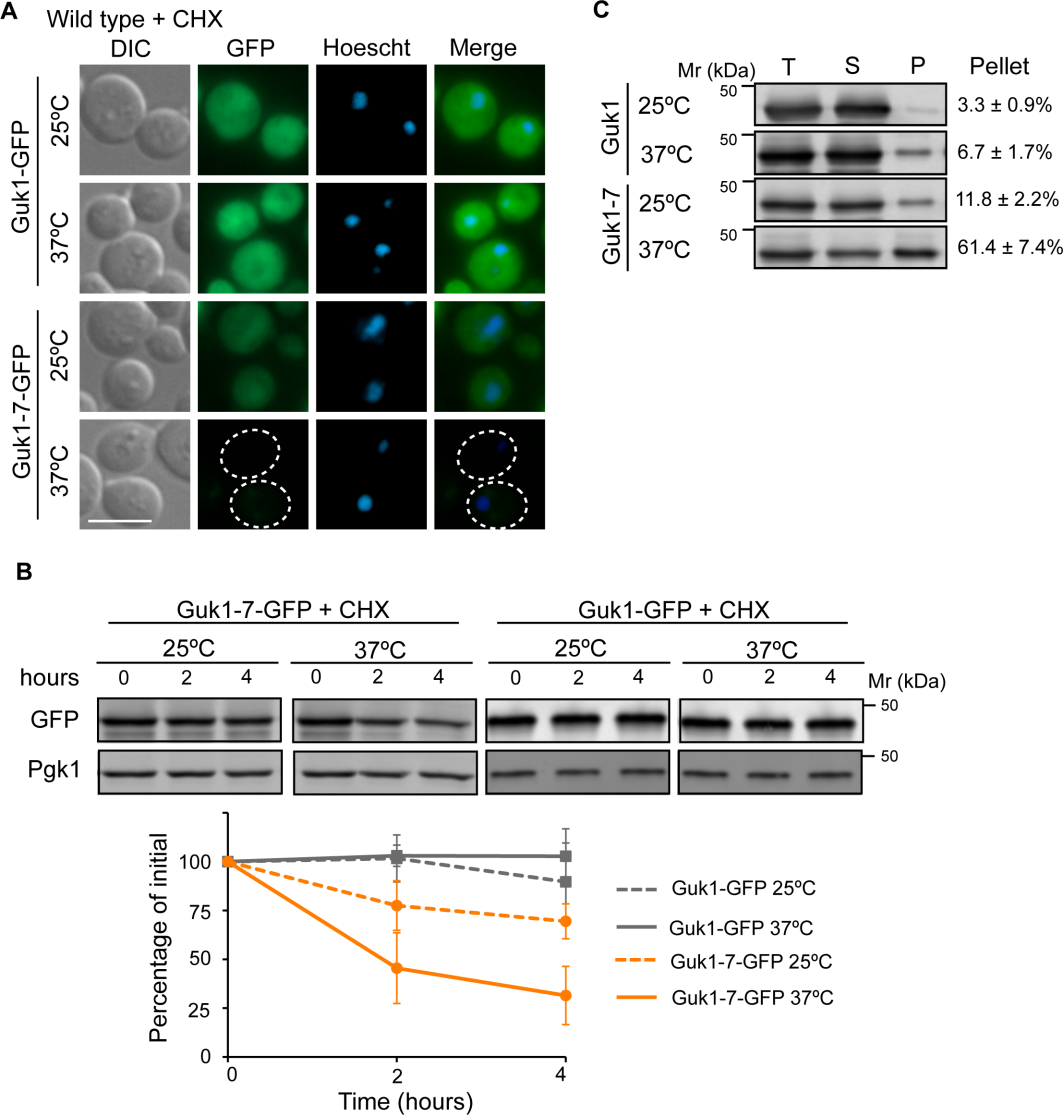
Misfolded Guk1-7 is degraded at the non-permissive temperature. (A) Wild type cells expressing ectopic Guk1-GFP or Guk1-7-GFP were grown at 25°C and then incubated in the presence of the translation inhibitor cycloheximide (CHX) at 25°C or 37°C for 2 hours prior to fixation and imaging. Scale bar represents 5μm. (B) Cycloheximide chase assay. Wild type cells expressing ectopic Guk1-GFP or Guk1-7-GFP were incubated with CHX for 4 hours at 25°C or 37°C and samples were collected at the indicated time points. Guk1-GFP and Guk1-7-GFP was immunoblotted with anti-GFP antibodies and a representative blot is shown. GFP levels were normalized to Pgk1 levels and shown in the graph below with results representing the means and standard deviations of three independent experiments. (C) Guk1-GFP and Guk1-7-GFP were ectopically expressed in wild type cells grown at 25°C or shifted to 37°C for 20 min. Total cell lysate (T), soluble (S), and pellet fractions (P) were immunoblotted with anti-GFP antibodies. The ratio of the pellet fraction to total cell lysate is noted and represents the mean and standard deviation of three independent experiments.

To determine whether the ectopically expressed mutant protein was also degraded when fused to GFP, we first examined fluorescence levels by microscopy. To determine whether the ectopically expressed mutant protein was also degraded when fused to GFP, we first examined fluorescence levels by microscopy. Guk1-7-GFP fluorescence was on average 58% lower than that of Guk1-GFP at 25°C (n = 101, 108), and was nearly undetectable with an average 87% loss of fluorescence following a two hour incubation at 37°C in the presence of the translation inhibitor cycloheximide (CHX) (n = 168) (Fig 2A, S1A Fig). In contrast, the fluorescence of the wild type Guk1-GFP only slightly decreased by 29% between 37°C and 25°C (n = 120). To verify that the loss of fluorescence was due to proteolysis and not misfolding of GFP, we examined levels of Guk1 and Guk1-7 by Western blot in a cycloheximide chase assay. While Guk1-GFP levels remained relatively unchanged, the level of Guk1-7-GFP decreased by 30% after a four hour incubation at 25°C, and decreased by 70% after the same period at 37°C (Fig 2B). We then verified whether adding a GFP tag alters Guk1 or Guk1-7 solubility. Three times as much Guk1-7 as Guk1 was found in the NP-40 insoluble pellet fraction at 25°C, and this rose to nine times more upon the short incubation at 37°C (Fig 2C). Although Guk1-7-GFP is less insoluble than Guk1-7-His6, presumably due to stabilization conferred by the GFP moiety, the GFP tagged mutant was both less soluble and more degraded than the wild type protein, and could therefore be employed as a model substrate.

**Fig 3:**
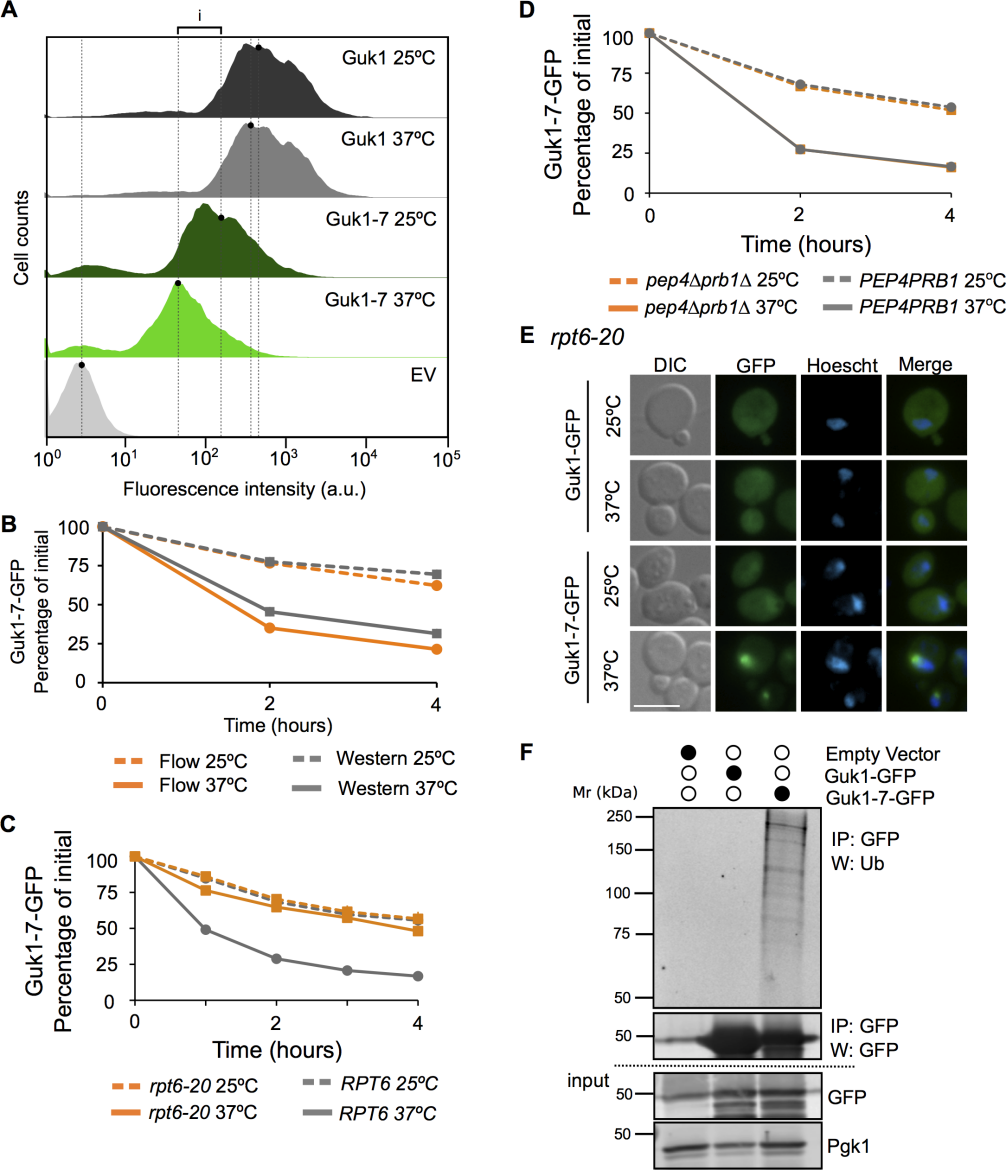
Guk1-7 degradation is proteasome dependent. (A) Flow cytometry profiles of wild type cells expressing Guk1-GFP or Guk1-7-GFP were incubated at 25°C or 37°C for two hours in the presence of CHX. Fluorescence in cells with the control empty vector (EV) are also shown. Lines demark median GFP fluorescence values and i denotes the difference in median intensity values used to measure protein stability. (B) Comparison of quantitation of Guk1-7 levels in a CHX chase assay by Western blot or flow cytometry. (C) Wild type and *rpt6-20* cells expressing Guk1-7-GFP were incubated with CHX at 25°C or 37°C and samples were analysed by flow cytometry at the indicated time points. The results represent the means and standard deviations of three independent experiments. (D) Guk1-7-GFP expressing wild type or *pep4∆prb1∆* cells were incubated at 25°C or 37°C in the presence of CHX and samples were analyzed by flow cytometry at the indicated time points. The results represent the means and standard deviations of three independent experiments. (E) *rpt6-20* cell expressing Guk1-GFP or Guk1-7-GFP were grown at 25°C and then shifted to 37°C for 1 hour prior to their fixation and imaging. Scale bar represents 5μm. (F) Guk1-GFP and Guk1-7-GFP expressing cells were incubated at 25°C and cell lysates were immunoprecipitated using GFP-Trap beads and then immunoblotted with anti-ubiquitin, anti-GFP, and anti-Pgk1 antibodies.

In order to use Guk1-7-GFP as a model substrate to screen for factors important in maintaining cytosolic protein homeostasis, we established a flow cytometry assay to monitor protein stability. Cultures were incubated at 25°C or 37°C in the presence of cycloheximide for two hours and then the GFP fluorescence intensity from single cells was measured by flow cytometry. The relative difference in median intensity values between 37°C and 25°C was used as a measure of protein stability. In a wild type strain at 25°C Guk1-7-GFP fluorescence intensity is lower than that of the wild type allele, suggesting that the model substrate is inherently unstable even at lower temperatures. After shifting the cells to 37°C in the presence of CHX for two hours, GFP intensity levels remained nearly constant for Guk1-GFP (5% loss) but decreased for Guk1-7-GFP (60% loss; Fig 3A). These data are consistent with our previous fluorescence microscopy and CHX-chase observations (Fig 2A and 2B). The data obtained from flow cytometry measurements was comparable to that acquired using traditional Western blotting techniques (Fig 3B).

The Guk1 and Guk1-7 constructs used in this report are ectopically expressed from the constitutive GPD promoter. To ensure that the overexpression from a plasmid does not influence the stability of our model substrate, we expressed both Guk1-GFP and Guk1-7-GFP from their endogenous locus and promoters, and performed a cycloheximide assay. Consistent with our previous CHX chase observations, Guk1-GFP levels remained relatively constant and Guk1-7-GFP levels decreased by approximately 30% after four hours at 25°C and by 60% when incubated at 37°C (S1B Fig). Together, this data suggested that the flow cytometry assay was suitable for monitoring protein levels and for screening purposes.

### Guk1-7 degradation is proteasome dependent

We next verified that degradation of the ectopically expressed model GFP-fusion substrate was proteasome-dependent. When we assayed levels of Guk1-7 at 37°C in the temperature sensitive proteasome mutant *rpt6-20* [19], degradation of the mutant protein was largely stopped (Fig 3C). Conversely, no difference in Guk1-7-GFP stability was seen between the wild type strain or a double mutant of the two main lysosomal proteases (Fig 3D). These results suggest that loss of Guk1-7-GFP fluorescence is primarily caused by proteasomal degradation. Fluorescence microscopy revealed that Guk1-GFP was evenly distributed with no inclusions in *rpt6-20* cells at both 25°C and 37°C, as was the case for Guk1-7-GFP at 25°C (n = 126, 104, 118, respectively) (Fig 3E). Guk1-7-GFP inclusions were detected in 69% of the cells incubated at 37°C (n = 120), of which 94% of cells contained a single inclusion and 5% contained two. These data indicate that non-degraded Guk1-7-GFP was prone to aggregation at the non-permissive temperature. Finally, we asked whether the difference in protein stability between Guk1-GFP and Guk1-7-GFP was also reflected by their respective ubiquitination levels. We found that Guk1-7-GFP, but not Guk1-GFP, was ubiquitinated at 25°C (Fig 3F). In this case, we collected cell lysates from cultures incubated at the lower growth temperature, as we encountered issues with our model substrate being mostly lost to the insoluble pellet fraction when cultures were grown at higher temperatures. Together these experiments suggest that misfolded Guk1-7 is targeted for degradation by the ubiquitin proteasome system.

### FACS-based screen for protein homeostasis factors

**Fig 4:**
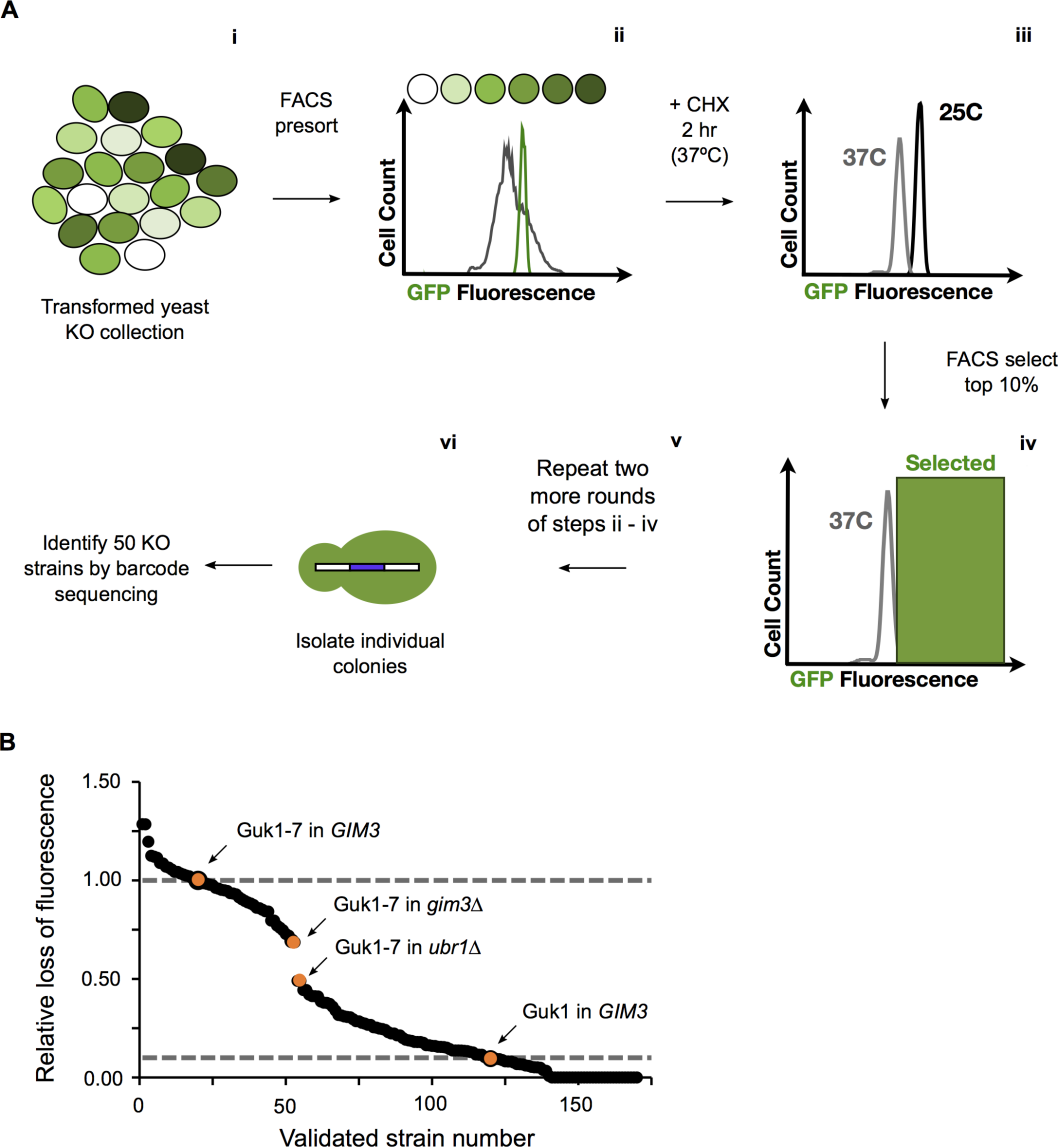
FACS-based screen. (A) Schematic of FACS-based screen. (B) Flow cytometry validation of 170 colonies isolated from the FACS screen. Relative loss of fluorescence of Guk1-GFP or Guk1-7-GFP in wild type cells is noted as a comparison.

To identify novel factors involved in targeting proteins destabilized by missense mutations for degradation, we performed a genome-wide screen based on flow cytometry using the Guk1-7-GFP allele. A schematic of the screen is depicted in Fig 4A. First, we pooled and bulk transformed the yeast non-essential knockout collection with a low copy number plasmid containing Guk1-7-GFP (Fig 4A i). Growth prior to and after transformation was limited, to avoid under representation of slow growing strains. Pooled transformants were grown in selective media at 25°C and then subjected to an initial fluorescence activated cell sorting (FACS) presort to obtain a narrow fluorescence range, which reduces cell-to-cell variability of GFP fusion expression (Fig 4A ii; compare grey and green profiles for before and after presort, respectively). Presorted cells were then incubated at 37°C in the presence of CHX for two hours (Fig 4A iii) and then sorted again, selecting for cells with GFP fluorescence in the top 10% range (Fig 4A iv). Samples were collected over a short fifteen minute period to minimize shifting of the population during the handling time. Cells were recovered in selective liquid media and the screen was repeated two more times for a total of three rounds of enrichment (Fig 4A v). Following the final FACS sorting, cells were collected on solid selective media plates (Fig 4A vi).

We selected 170 colonies, which had been isolated using the FACS screen described above, for validation using the flow cytometry assay. In approximately two thirds of the colonies tested, Guk1-7-GFP was more stable than in the wild type cells (Fig 4B). Fifty colonies were then selected at random, spanning the range of Guk1-7-GFP stabilities, and their corresponding gene knockouts were identified by Sanger sequencing of the unique strain-specific barcodes. From these fifty colonies, we identified fifteen different gene deletions (Table 1). Next, to ensure that the phenotype (*i*.*e*., stabilization of Guk1-7) was not acquired during the screening process, we assessed the stability of Guk1-7-GFP for each of the fifteen gene deletions we identified through Sanger sequencing. To do so, we individually retransformed the Guk1-7-GFP containing plasmid into each knockout strain from our pre-pooled knockout collection. Results of this phenotypic validation were considered positive (denoted as P in Table 1) if Guk1-7-GFP was at least 15% more stable in the deletion strain than in wild type cells (S1C Fig). Strains that did not meet this criterion were classified as negative (denoted as N in Table 1). Surprisingly, we failed to observe any stabilization of our model substrate in our most frequently identified hits (*e*.*g*., *tda2∆*), which may have been susceptible to the acquisition of secondary mutations (Table 1). Among the validated hits, we identified the N-end rule E3 ligase Ubr1, which was previously shown to target cytosolic misfolded proteins for degradation [14–18, 26], and the prefoldin chaperone subunit Gim 3 (Table 1, S1C Fig).

**Table 1:** Summary of FACS screen validation.

Standard Name	Systematic Name	Number of Times Barcode Identified by Sanger Sequencing	Result of Phenotypic Validation^a^
TDA2	YER071C	22	N
YAK1	YJL141C	10	N
RIM15	YFL033C	5	P
UBR1	YGR184C	2	P
YOR364W	YOR364W	1	P
GIM3	YNL153C	1	P
SLI15	YBR156C	1	N
FAU1	YER183C	1	N
VHR1	YIL056W	1	N
BRA7	YER056C	1	P
ASP1	YDR321W	1	N
MAL11	YGR289C	1	N
MUP3	YHL036W	1	P
POL4	YCR014C	1	N
PPH22	YOL188C	1	N

^a^P: positive, N: negative.

### Ubr1 stabilizes Guk1 missense mutant

**Fig 5:**
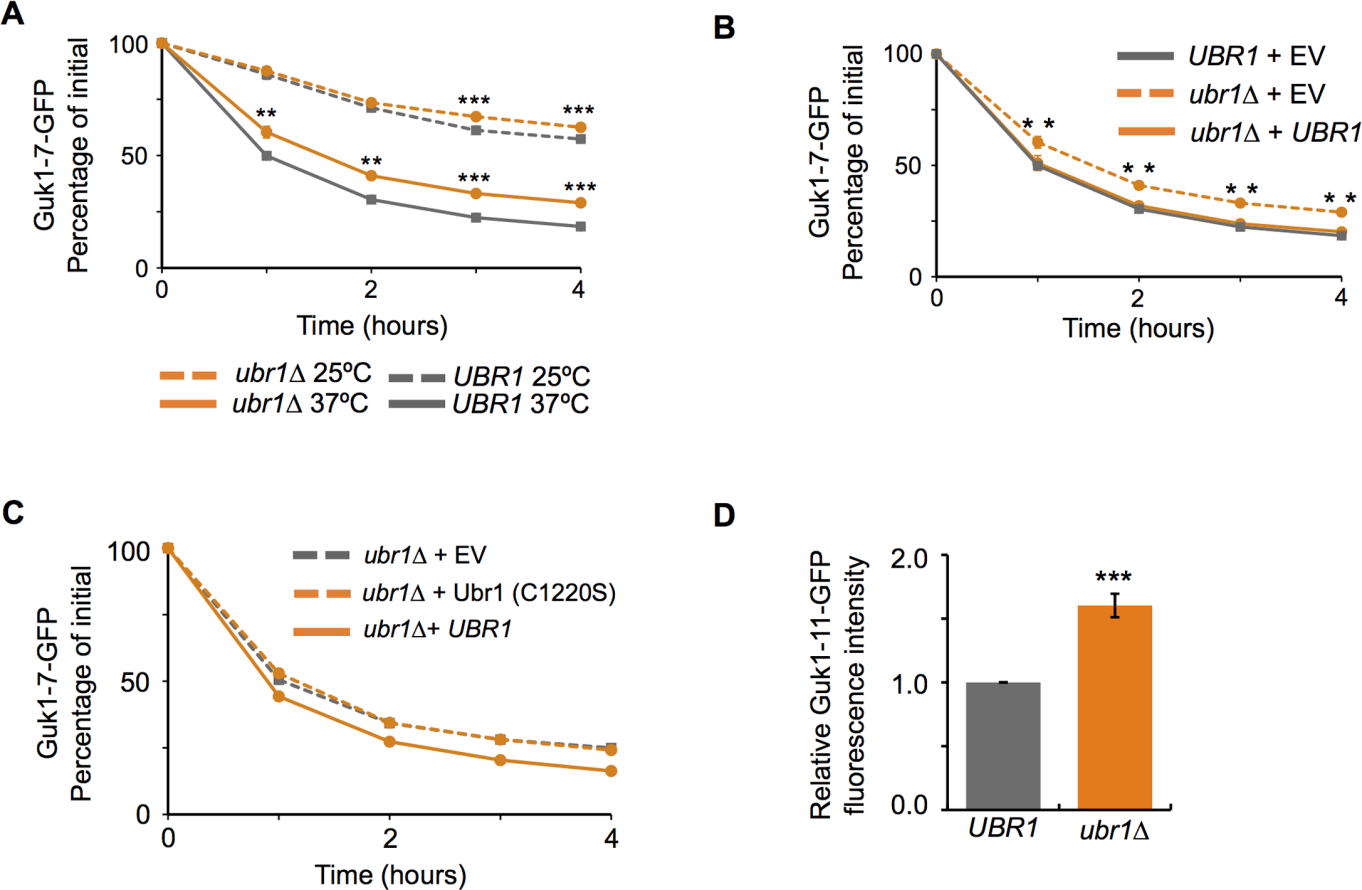
Ubr1 promotes Guk1-7-GFP degradation. (A) Wild type and *ubr1∆* cells expressing Guk1-7-GFP were incubated with CHX at 25°C or 37°C and samples were analysed by flow cytometry at the indicated time points. The results represent the means and standard deviations of three independent experiments. P values were calculated with an unpaired Student’s t test, *, ** and *** denote P < 0.05, 0.005, and 0.0005, respectively. (B) *UBR1* and *ubr1∆* cells expressing Guk1-7-GFP along with an empty vector (EV) control or *UBR1* were incubated at 37°C and samples were collected at the indicated time points for flow cytometry analysis. Results represent the means and standard deviations of three independent experiments. P values were calculated with a one-way ANOVA and post-hoc Tukey HSD to assess significance, ** denotes P < 0.005. (C) *ubr1∆* cells coexpressing Guk1-7-GFP and an empty vector control or either *UBR1* or *UBR1* (C1220S) were incubated at 37°C with CHX and samples were collected at the indicated time points. (D) Wild type or *ubr1∆* cells expressing Guk1 (T290G) fused to GFP were incubated with CHX at 37°C for two hours before being analyzed by flow cytometry. The results represent the relative fluorescence intensities from three independent experiments (with standard deviations). P values were calculated with a one-way ANOVA and post-hoc Tukey HSD to assess significance, *** denotes P < 0.0005.

We identified the E3 ubiquitin ligase Ubr1 in our screen for factors responsible for degradative protein quality control of misfolded cytosolic proteins destabilized by missense alleles. In addition to its role as the E3 ligase of the N-end rule pathway, Ubr1 has also been shown to target misfolded cytoplasmic proteins for degradation [15, 18, 26]. In CHX chase experiments, Guk1-7-GFP levels were approximately 10% higher in *ubr1∆* cells compared to wild type (P = 0.0008) at two hours and remained significantly higher after four hours (P = 0.00003) (Fig 5A). To confirm that the stability observed was directly caused by the loss of Ubr1, we performed addback experiments whereby the wild type Ubr1 was expressed from a plasmid in *ubr1∆* cells. We observed that Guk1-7-GFP levels were similar between the *UBR1* cells containing a control empty vector and *ubr1∆* cells with the Ubr1 expressing plasmid, confirming that the phenotype observed could be attributed to the absence of Ubr1 (Fig 5B). To further validate our findings, we performed the same addback experiments but this time included a mutant form of Ubr1, which contains a point mutation in the RING domain producing an inactive ligase [15]. Guk1-7-GFP levels in the Ubr1 (C1220S) expressing cells were indistinguishable from those with a control plasmid, lending further support to Ubr1 having a direct role in controlling Guk1-7 stability (Fig 5C). We next assessed the importance of Ubr1 on a second unstable allele of Guk1 (T290G) that contained a single missense mutation. This mutant was generated by site directed mutagenesis and was selected based on its instability, as a two hour incubation at 37°C in the presence of cycloheximide typically resulted in approximately 60% loss of fluorescence. Consistent with our previous results, an absence of *UBR1* led to a significantly reduced clearance of this second unstable model substrate (P = 0.00035) (Fig 5D). The relative fluorescence of Guk1-GFP was not significantly different between wild type and *ubr1∆* cells (S2A Fig). These results indicate Ubr1 participates in the clearance of these model misfolded substrates, although other factors are also involved.

Ubr1 has been shown to act in concert with the nuclear E3 ligase San1 to target misfolded cytoplasmic proteins for degradation [15, 26]. To test whether Ubr1 also acts with San1 in the degradation of Guk1-7-GFP, we performed flow cytometry experiments in the single *ubr1∆* and *san1∆* deletion strains along with a double *ubr1∆san1∆* deletion. Guk1-7-GFP was not markedly more stable upon the deletion of *SAN1*, although levels were slightly higher in *ubr1∆san1∆* cells in comparison to *ubr1∆* cells (S2B Fig). These results indicate that San1 does not play a major role in the turnover of Guk1-7. To confirm that our assay was capable of detecting an effect with San1, we ran the same assay using the previously characterized Ubr1 and San1 substrate Pro3-1 [26]. In this case we were able to observe a significant stabilization of Pro3-1 in *san1∆* cells, which was even more pronounced in the double *ubr1∆san1∆* strain (S2C Fig). Together, these data suggest that San1 does not play a role alongside Ubr1 in targeting Guk1-7-GFP for degradation, indicating that other E3 ligases may be involved in the proteasome-mediated degradation of this substrate.

### Gim3 impairs Guk1-7-GFP degradation

**Fig 6:**
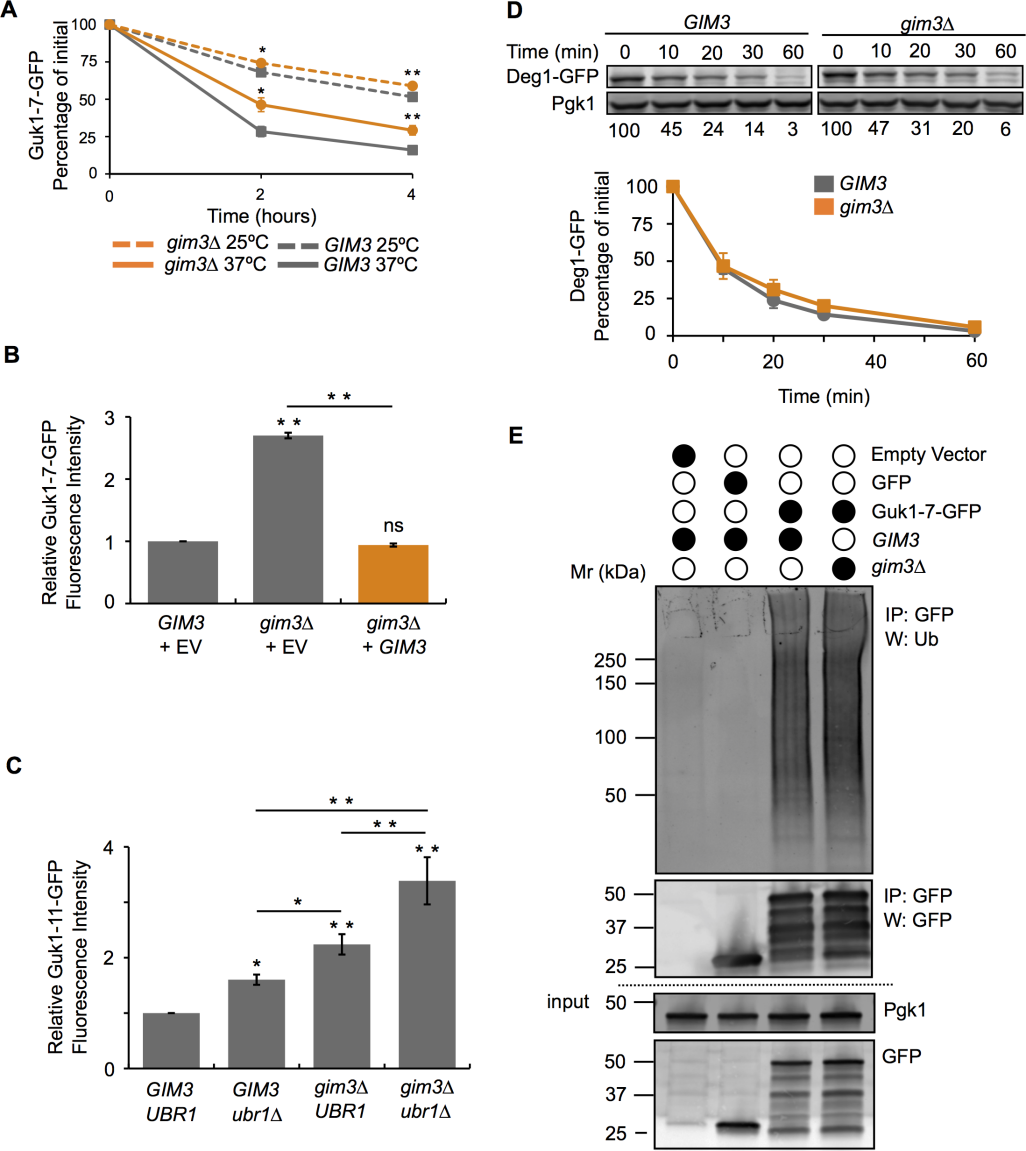
Absence of Gim3 reduces Guk1-7 turnover. (A) Wild type and *gim3∆* cells expressing Guk1-7-GFP were incubated with CHX at 25°C or 37°C and samples were analysed by flow cytometry at the indicated time points. The results represent the means and standard deviations of three independent experiments and the asterisk denotes significance of P < 0.05. (B) Gim3 addback experiment. *GIM3* or *gim3∆* cells expressing Guk1-7-GFP and either an empty vector (EV) control or *GIM3*. The results represent the means and standard deviations of three independent experiments of the relative fluorescence intensity after a two hour CHX incubation at 37°C. P values were calculated with a one-way ANOVA and post-hoc Tukey HSD to assess significance, ** denotes P < 0.005. (C) Wild type, *ubr1∆*, *gim3∆*, and *ubr1∆gim3∆* cells expressing Guk1 (T290G) fused to GFP were incubated at 37°C with CHX for two hours and then analysed by flow cytometry. P values were calculated with a one-way ANOVA and Holm multiple comparison to assess significance, * and ** denote P < 0.05 and 0.01, respectively. (D) Proteasome activity assay. *Gim3* or *gim3∆* cells expressing Deg1-GFP under the Cup1 promoter were incubated at 30°C in the presence of CHX and samples were collected at the indicated time points. Deg1-GFP was immunoblotted with an anti-GFP antibody. The results represent the mean and standard deviation of three independent experiments. (E) *GIM3* or *gim3∆* cells expressing Guk1-7-GFP, GFP alone, or a control empty vector were grown at 25°C. Guk1-7-GFP was immunoprecipitated with GFP-Trap beads and eluted samples were immunoblotted with anti-ubiquitin and anti-GFP antibodies.

Prefoldin is a hetero-oligomeric protein complex composed of six subunits ranging in size from 14–23 kDa [36]. Conserved in archaea and eukaryotes, but absent in prokaryotes, the prefoldin hexamer forms a “jellyfish-like” structure with N- and C-terminal coiled-coil regions of each subunit forming “tentacles” that emanate from a central region [37]. Misfolded substrates are transferred for folding from prefoldin to the TRiC/CCT chaperonin in an ATP-independent manner through direct binding of the two chaperone complexes [36]. In addition to its role in aiding nascent proteins, such as actin and tubulin, to attain their functional conformations, the prefoldin chaperone complex has been shown to prevent huntingtin and alpha-synuclein aggregate formation [38, 39]. Having identified the prefoldin subunit Gim3 in our screen, we decided to further examine its potential role in degradative protein quality control. In CHX chase experiments, Guk1-7-GFP levels were approximately 25% higher in the *gim3∆* strain compared to wild type (P = 0.0057) (Fig 6A). To ensure that the stabilization was specifically caused by the absence of Gim3, we expressed in *gim3∆* cells the wild-type *GIM3* from a plasmid, which rescued the degradation of the model substrate (Fig 6B). While degradation of Guk1-7-GFP is not fully inhibited in *gim3∆* cells, levels are markedly higher than in the wild type strain, indicating that Gim3 is required for the normal turnover of our model substrate. We next wished to see if Gim3 works together with Ubr1. In this case we preferred a model substrate that is misfolded as the result of a single point mutation (Guk1 (T290G)) to eliminate or minimize potential confounding factors caused by multiple destabilizing mutations. The double *ubr1∆gim3∆* strain showed increased Guk1 (T290G) stability compared to single deletion strains, however the substrate was still degraded by over 50% (Fig 6C). This data indicates that potentially other E3 ligases or chaperones are required for complete proteolysis to occur. In addition, this would suggest that Ubr1 and Gim3 work partially in parallel or in independent pathways to target the assessed misfolded substrate for degradation.

The TRiC/CCT chaperonin cooperates with prefoldin in folding a number of cellular proteins and has been shown to interact with proteasome subunits, suggesting that it may be involved in proteasome maturation [40]. Therefore, one possibility is that the stabilizing effect of Gim3 on Guk1-7 could be indirect, a result of decreased proteasome function. We tested for compromised proteasome function in the *gim3∆* strain using the constitutive Deg1-GFP proteasome substrate [41]. We found that there was no significant difference in the degradation of Deg1-GFP in *gim3∆* cells compared to the wild type strain at all time points tested, with the exception of the thirty minute sample (P = 0.77, P = 0.22, P = 0.04, P = 0.07 for the 10, 20, 30, and 60 minute time points, respectively) (Fig 6D). Hence, the reduced turnover of misfolded protein observed in *gim3∆* cells is unlikely caused by an impaired proteasome.

We next determined whether an absence of *GIM3* could affect ubiquitination of our misfolded model substrate. GFP tagged Guk1-7 was pulled down from cells grown at 25°C, where it remained mostly soluble, and then ubiquitin levels were detected by immunoblotting. After normalizing the quantity of ubiquitin to that of eluted Guk1-7, ubiquitination levels were essentially unchanged, with under 5% less ubiquitinated model substrate in *gim3∆* cells compared to wild type (Fig 6E). This experiment indicates that the absence of Gim3 did not impair ubiquitination of our model substrate, in agreement with Gim3 functioning independently of Ubr1.

### Gim3 facilitates the clearance of insoluble Guk1 and maintains Guk1-7 solubility

**Fig 7:**
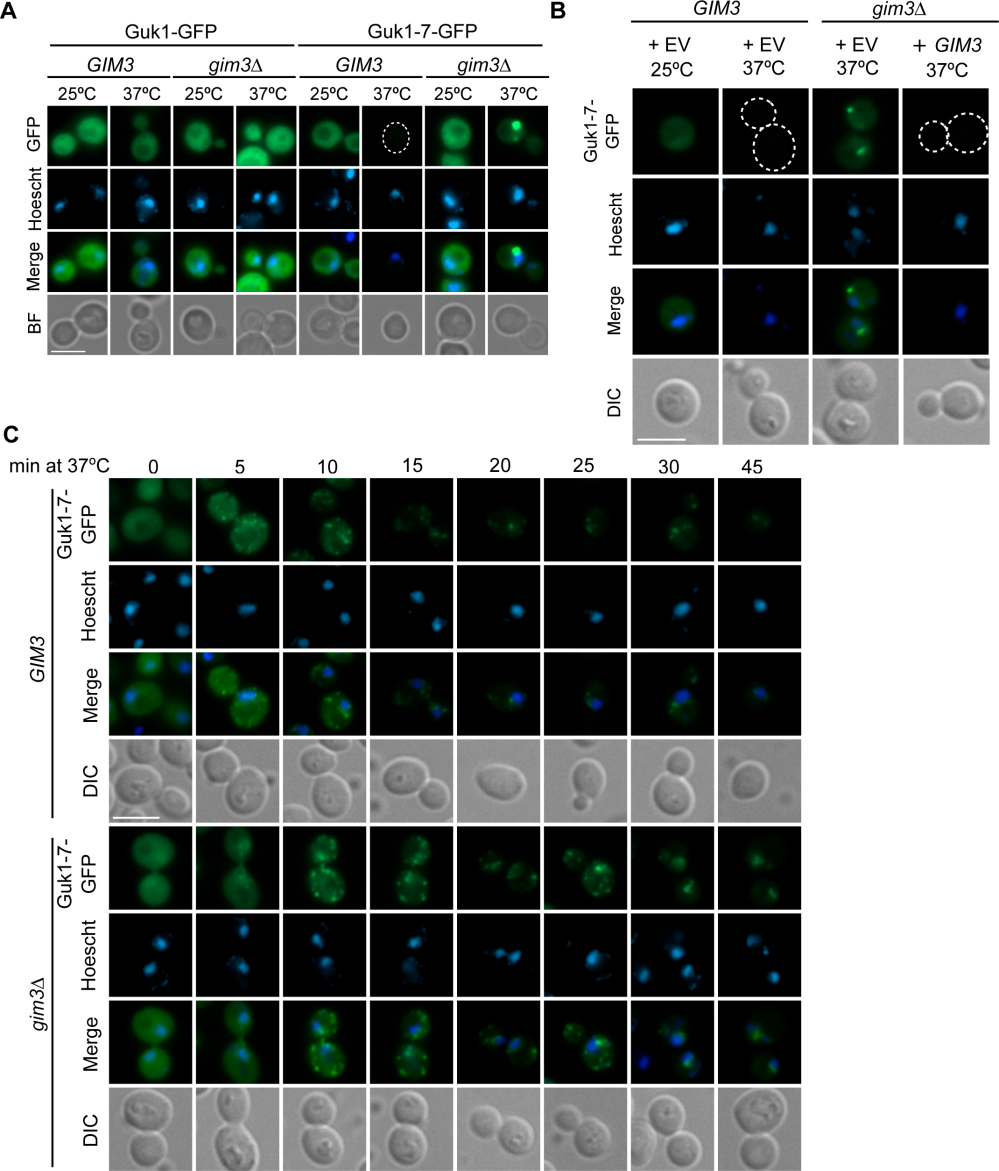
Gim3 facilitates clearance of insoluble Guk1-7. (A) *GIM3* or *gim3∆* cells expressing Guk1-7-GFP were grown at 25°C and then incubated at either 25°C or 37°C for 2 hours in the presence of CHX before fixation and imaging. (B) *GIM3* and *gim3∆* cells expressing Guk1-7-GFP along with an empty vector control or *GIM3* were incubated at 25°C or 37°C for two hours in the presence of CHX before fixation and imaging. (C) *GIM3* or *gim3∆* cells expressing Guk1-7-GFP were incubated at 25°C and then shifted to 37°C. Samples were collected at the indicated time points and then fixed before imaging. For all images, the scale bar represents 5μm and dotted lines demark cell boundaries.

We next sought to evaluate the impact an absence of Gim3 has on Guk1-7 localization. Fluorescence microscopy performed on wild type and *gim3∆* strains showed that while there was no difference in wild type Guk1-GFP localization between the two strains, Guk1-7-GFP formed cytoplasmic puncta in 93% of *gim3∆* cells when incubated at 37°C, and additional faint and diffuse cytoplasmic GFP was also visible (n = 200) (Fig 7A). Cells contained on average 1.5 puncta, which were typically located next to the nucleus. In contrast, only sixteen percent of wild type cells contained Guk1-7-GFP puncta (n = 200). These puncta were no longer present when we expressed Gim3 from a plasmid in *gim3∆* cells and the diffuse cytoplasmic GFP signal was also not present, similar to that observed in *GIM3* cells (Fig 7B). We decided to examine this phenomenon more closely by performing a time course microscopy experiment incubating cells at 37°C, but in the absence of the translation inhibitor CHX which we had been using up to this point and which may interfere with aggregate formation [42]. Within five minutes numerous Guk1-7-GFP containing puncta were detected within the cytoplasm of *GIM3* cells with those in the *gim3∆* strain being slightly delayed and visible after 10 minutes (Fig 7C). While puntca remained visible in both strains after 45 minutes at 37°C, those in the *gim3∆* strain appeared to coalesce slower than in *GIM3* (after 15–20 minutes in *GIM3* compared to 20–25 minutes in *gim3∆*) and remained visibly brighter. Some diffuse cytoplasmic Guk1-7-GFP signal was also present in *gim3∆* cells up to 25 minutes after shifting to the increased growth temperature, but was only present in the first 10 minutes for *GIM3* cells. While the number of puncta did not differ between Gim3 containing or deleted cells, the intensity of the *gim3∆* puncta remained brighter for longer. These results suggest that Gim3 may play a role in maintaining Guk1-7-GFP solubility at higher temperatures to facilitate substrate degradation.

**Fig 8:**
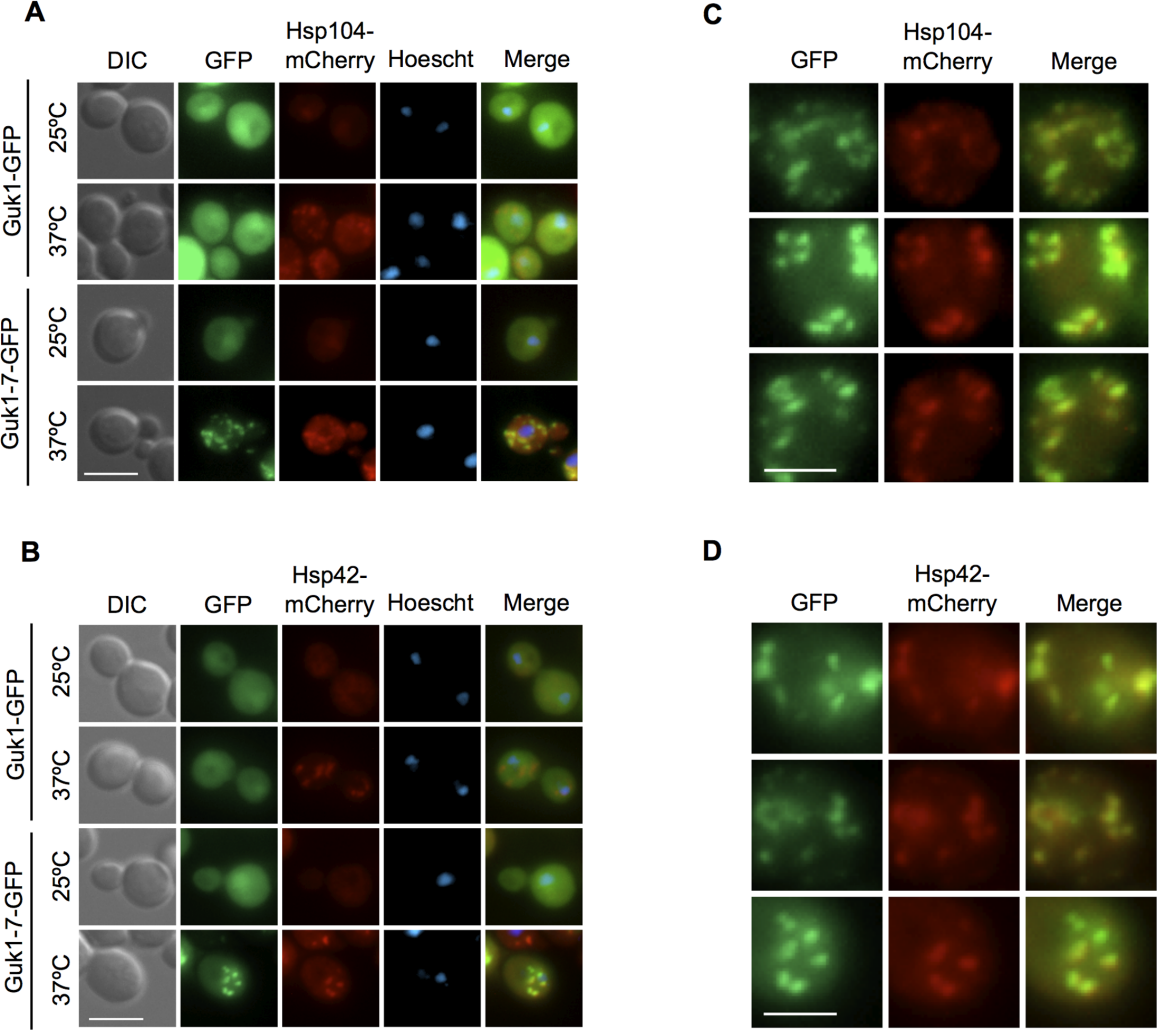
Guk1-7-GFP puncta colocalize with Q-body markers. (A) Cells with Hsp104 endogenously tagged with mCherry and ectopically expressing Guk1-GFP or Guk1-7-GFP were grown at 25°C and then incubated at 25°C or 37°C for 30 minutes before fixation and imaging. Scale bar represents 5μm. (B) Hsp42-mCherry cells ectopically expressing Guk1-GFP or Guk1-7-GFP were grown at 25°C prior to incubation at 25°C or 37°C for 30 minutes. Cells were then fixed before imaging. The scale bar represents 5μm. (C) Enlarged images from cells collected as in A. Scale bar represents 2.5μm. (D) Enlarged images from cells collected as in B. Scale bar represents 2.5μm.

These Guk1-7-GFP puncta observed in the time course experiment in both *GIM3* and *gim3∆* cells are reminiscent of the Q-bodies described by Frydman and colleagues [22]. To determine whether this is indeed the case, we examined Guk1-GFP and Guk1-7-GFP colocalization in *GIM3* cells with two cytosolic aggregate markers: Hsp104 and Hsp42. Hsp104 is an aggregate-specific chaperone that has a diffuse cytoplasmic and nuclear localization pattern at 25°C but forms puncta when incubated at 37°C (Fig 8A, enlarged Fig 8C). Hsp104-mCherry and Guk1-7-GFP puncta colocalized in 100% of cells at 37°C (n = 100). We then examined Guk1-7-GFP colocalization with the small heat shock protein Hsp42, which is required for peripheral aggregate formation during physiological heat stress [43]. As with Hsp104, Hsp42-mCherry formed puncta when incubated at 37°C, but not at 25°C (Fig 8B, enlarged Fig 8D). Guk1-7-GFP colocalized in all Hsp42-mCherry puncta. However, in 34% of the cells examined (n = 100), we find an average of 1.4 Guk1-7-GFP puncta per cell that do not colocalize with Hsp42. Overall, Hsp42-free Guk1-7-GFP puncta represented 9% of all puncta observed in the one hundred cells examined. Together, the Hsp104 and Hsp42 colocalization data suggest that at 37°C Guk1-7-GFP forms cytosolic inclusions similar to Q-bodies.

**Fig 9:**
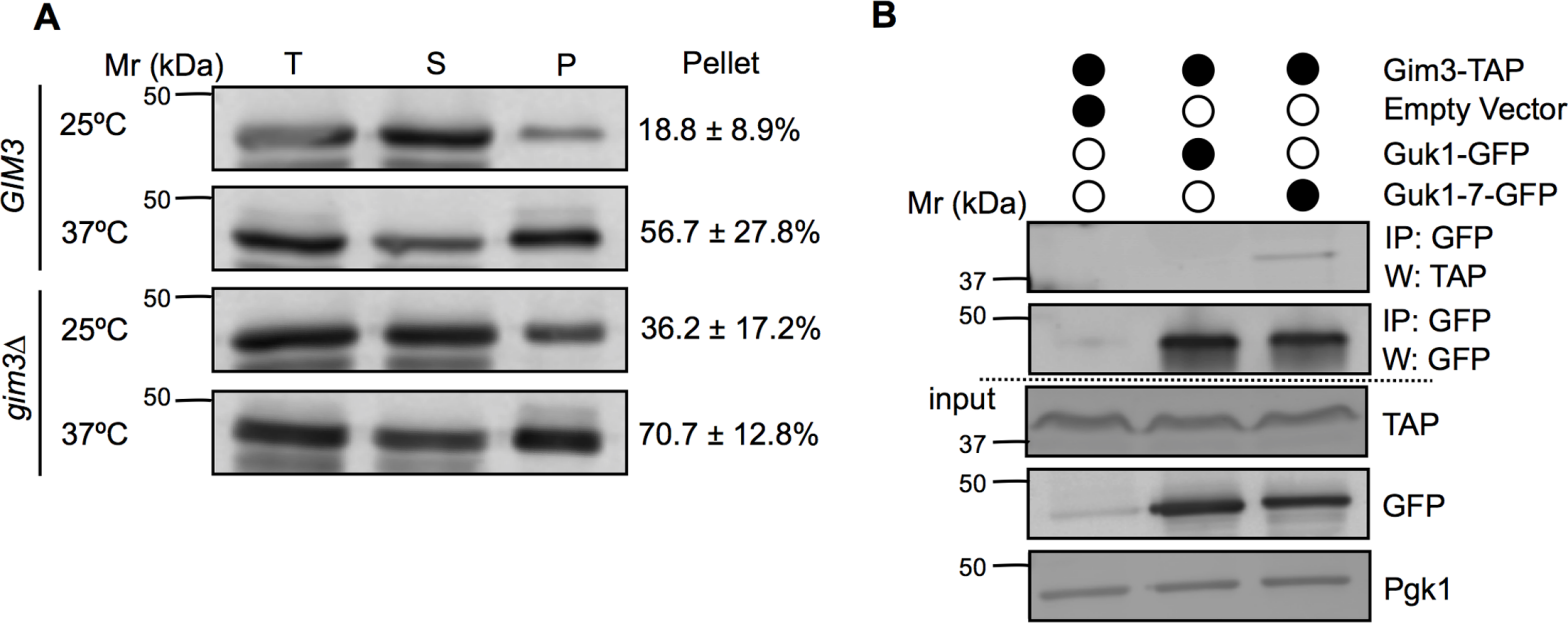
Gim3 helps maintain Guk1-7 solubility. (A) *GIM3* or *gim3∆* Guk1-7-GFP expressing cells were grown at 25°C or shifted to 37°C for 20 min. The ratio of the pellet fraction to total cell lysate is noted and represents the mean and standard deviation of three independent experiments. (B) Guk1-7-GFP was immunoprecipitated from Gim3-TAP expressing cells incubated at 25°C and then immunoblotted with anti-TAP, anti-GFP, or anti-Pgk1 antibodies.

To verify the importance of Gim3 in maintaining Guk1-7 solubility, we examined the sedimentation of the mutated protein after centrifugation. Twice as much Guk1-7 is found in the NP-40 insoluble pellet fraction at 25°C in *gim3∆* cells compared to wild type *GIM3* cells (Fig 9A). There was also more Guk1-7 in the pellet of *gim3∆* than wild type cells after incubating cells at 37°C. We then performed immunoprecipitation experiments to test whether Gim3 could directly interact with Guk1-7 as a potential mechanism for maintaining Guk1-7 solubility. From cell extracts incubated at 25°C, Guk1-7-GFP can pull down a TAP-tagged form of Gim3 whereas no interaction was detected between Gim3-TAP and Guk1-GFP (Fig 9B). We verified this interaction in an independent experiment (S3A Fig). Once again, a lower temperature was used for pulldown experiments to avoid losing Guk1-7 in the insoluble pellet fraction. These results suggest that Gim3 could maintain Guk1-7 in a more soluble state through physical interaction, potentially acting as a holdase. Holdases are a type of molecular chaperone that bind to misfolded proteins in an ATP-independent manner to prevent protein aggregation, but they do not directly refold their substrates [44]. Consistent with these findings, we tested the viability of the *guk1-7* strain over a range of temperatures (25°C to 37°C), in the presence or absence of *GIM3*, and found that in both cases viability largely decreased between 32°C and 33°C with no growth at temperatures of 34°C or above (S3B Fig). These results indicate that, whereas degradation of poorly soluble Guk1-7 was delayed, temperature-dependent lethality is not rescued in *gim3∆* cells.

### Gim3 has a general effect towards thermally destabilized proteins

**Fig 10:**
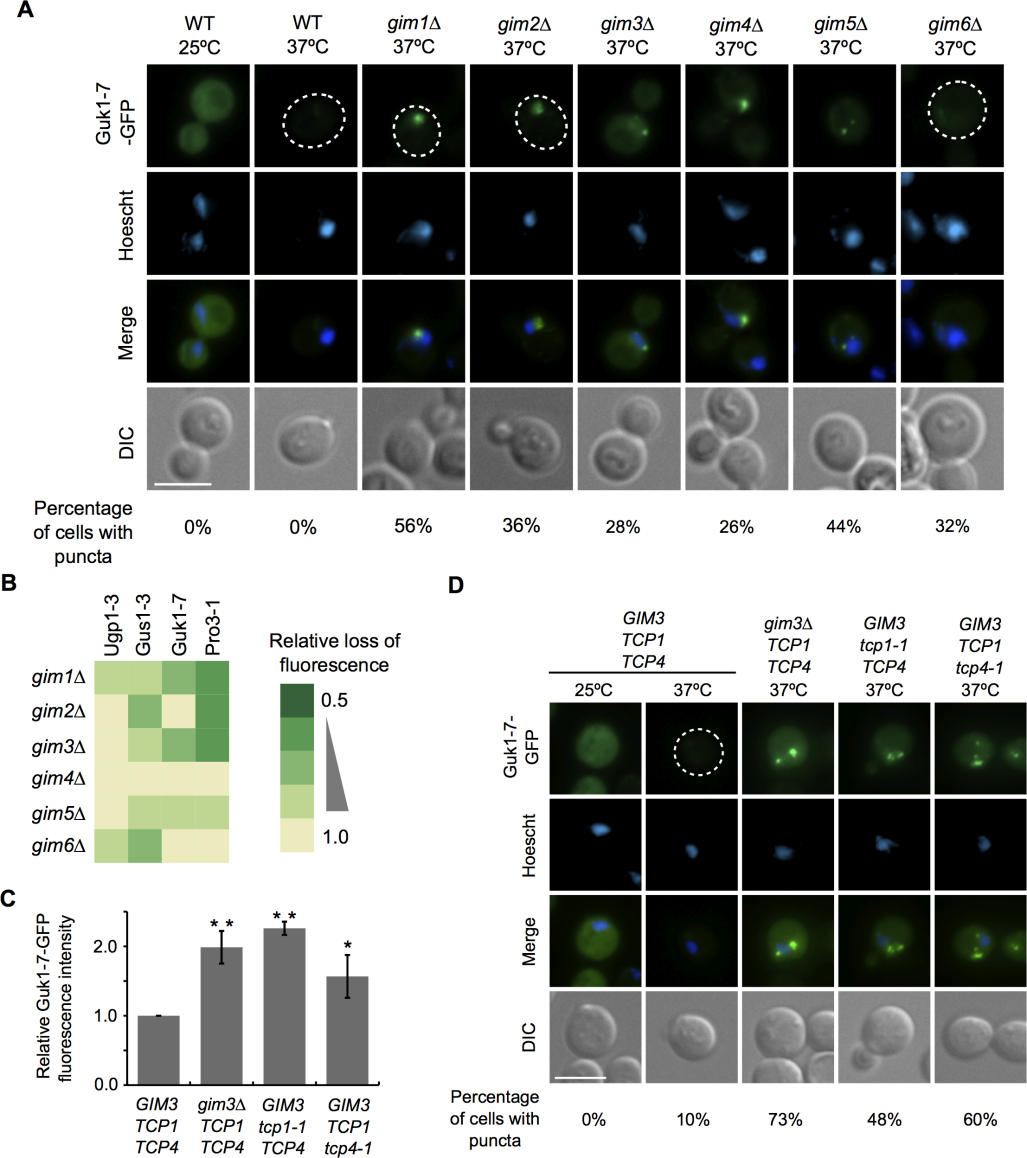
Thermosensitive alleles are stabilized by prefoldin subunits. (A) Individual prefoldin subunit mutants expressing Guk1-7-GFP were incubated at 37°C in the presence of CHX for 2 hours. The percentage of cells with puncta was calculated from 30–50 GFP positive cells. Scale bar represents 5μm. (B) Four thermosensitive alleles were expressed as GFP fusion proteins in the six prefoldin deletion strains. Cells were incubated with CHX at 25°C and 37°C for 2 hours and fluorescence intensity was measured by flow cytometry. (C) Guk1-7-GFP was expressed in wild type, *gim3∆*, *tcp1-1*, or *tcp4-1* cells and incubated with CHX for two hours at either 25°C or 37°C before flow cytometry analysis. (D) Wild type, *gim3∆*, *tcp1-1*, and *tcp4-1* cells expressing Guk1-7-GFP were incubated with CHX for 2 hours at 25°C or 37°C prior to fixation and imaging. Scale bar represents 5μm.

To see if the effect of Gim3 on Guk1-7 solubility was specific to this prefoldin subunit, or common to all prefoldin subunits, we performed fluorescence microscopy with the other prefoldin mutant strains. Guk1-7-GFP puncta were visible in all of the prefoldin deletions, albeit to varying degrees, suggesting that they all play a role in Guk1-7 solubility (Fig 10A). Fifty-six per cent of *gim1∆* cells contained puncta, whereas only 26% of *gim4∆* did (n = 50, each). While the number of puncta per cell only differed slightly between prefolin strains, either faint or no cytoplasmic Guk1-7-GFP was visible in *gim2∆*, *gim4∆*, *gim5∆*, and *gim6∆* strains while markedly present in *gim1∆* and *gim3∆* cells. To better quantify the effect, we measured Guk1-7 levels by flow cytometry and found that only deletions of *GIM1* and *GIM3*, and to a lesser extent *GIM5*, retarded the degradation of the model substrate (Fig 10B). Not surprisingly, *gim1∆* and *gim3∆* were the only strains that, in addition to puncta, also had a diffuse cytoplasmic Guk1-7-GFP signal visible by fluorescence microscopy. All together, these results suggest that while deletion of individual members of the prefoldin complex impacted degradation of the model substrate, some (*i*.*e*., Gim1 and Gim3) may play a more important role.

We next wished to explore whether prefoldin can stabilize additional proteins that are destabilized by missense mutations other than Guk1-7. We selected a number of cytoplasmic thermosensitive alleles, which we have previously shown to be degraded in a proteasome dependent manner, and created C-terminal GFP fusions to test by flow cytometry [26]. Ugp1 is the UDP-glucose pyrophophorylase in *S*. *cerevisiae* and is involved in the oxidative stress response [45, 46]. The Ugp1-3 allele contains two silent and two missense mutations and temperature sensitive lethality can be restored by Ubr1 deletion [26]. Ugp1-3-GFP was only modestly stabilized by all prefoldin deletions (including *gim3∆*) with the largest effect seen in *gim1∆* and *gim6∆* strains (Fig 8B). Glutamyl tRNA synthetase (Gus1) attaches amino acids to cognate tRNA and the Gus1-3 allele contains seven missense mutations [26, 47, 48]. Unlike Guk1-7, Gus1-3 was most stabilized by *gim2∆* and *gim6∆* strains. Delta 1-pyrroline-5-carboxylate reductase (Pro3) converts delta 1-pyrroline-5-carboxylate to proline in the final step of the proline biosynthesis pathway [49]. Pro3-1 has four missense mutations and its temperature sensitive lethality is restored in a double deletion of Ubr1 and San1 [26]. Of all the alleles tested, Pro3-1 was the most stabilized by the prefoldin deletions, with *gim1∆*, *gim2∆*, and *gim3∆* strains having the largest effect. Gim5 also stabilized Pro3-1, but to a lesser extent. The *gim4∆* strain had the smallest effect on substrate stabilization of all the deletions tested. These data suggest that while individual prefoldin subunit deletions may differentially affect substrate stability, they can stabilize a range of substrates misfolded due to missense mutations.

Given that the TRiC/CCT chaperonin folds client proteins delivered to it by the prefoldin complex, we sought to see if it also had a role in stabilizing our Guk1-7-GFP substrate. Using temperature sensitive alleles of the TRiC/CCT subunits Tcp1 and Tcp4, we performed CHX chase experiments and found Guk1-7-GFP levels to be significantly higher compared to wild type, similar to those found in *gim3∆* cells (Fig 10C). Fluorescence microscopy showed that Guk1-7-GFP forms cytoplasmic puncta in 48% of *tcp1-1* cells (n = 100) and 60% of *tcp4-1* cells (n = 100) when incubated at 37°C (Fig 10D). In cells where puncta were observed, an average of 1.8, 2.4, and 1.3 puncta per cell were found for the *gim3∆*, *tcp1-1*, and *tcp4-1* strains, respectively. These data would suggest a possible role for TRiC/CCT chaperonin in addition to prefoldin in maintaining the solubility of our Guk1-7-GFP model substrate.

## Discussion

Classically, degradative quality control pathways have been identified and characterized using model substrates. In this study, we have established Guk1-7 as a novel model protein quality control substrate whose stability is temperature dependent and is degraded by the proteasome. The mutant protein also forms Q-body like inclusions when shifted to the non-permissive temperature that co-localize with Hsp104, as well as Hsp42. We developed a flow cytometry assay to assess protein stability and then performed a FACS-based screen to isolate factors important for cytosolic protein homeostasis. We identified the E3 ubiquitin ligase Ubr1 and the prefoldin chaperone subunit Gim3. Gim3 promotes Guk1-7-GFP degradation and influences its solubility, but not ubiquitination. We also showed that in addition to Guk1-7, prefoldin can stabilize a number of temperature sensitive proteins that misfold as the result of missense mutations.

Protein degradation is generally assayed by pulse-chase metabolic labelling, or by using protein synthesis inhibitors coupled with downstream biochemical analysis [50]. More recently, fluorescently tagged proteins have been used to monitor protein stability [15, 51]. This development means that it is now feasible to perform high-throughput genome-wide screens using flow cytometry to identify factors that influence protein stability or abundance. Flow cytometry confers a number of advantages compared to stability assays using endogenous or ectopically expressed fluorescently tagged substrates as the method is quantitative, measurements are performed *in vivo*, thousands of cells can be analyzed in under an hour, and for most purposes no additional processing or cell lysis is required. While temperature sensitive alleles have been used in suppressor screens to identify protein quality control components such as San1 and Ubr1, these screens require that the model substrate be functional at the restrictive temperature [12, 26]. Perhaps most importantly, in addition to the relative speed and precision flow cytometry provides over Western blotting methods, the assay is sensitive enough to discern partial effects (e.*g*., stabilization of Guk1-7 in *ubr1∆*) that might not be detected by traditional Western blotting methods and can be used as a screening tool that does not rely on protein function.

Limiting the damaging effects of misfolded proteins appears to have influenced protein evolution as the most conserved proteins are those with the highest translation rate and as a result face the greatest risk of incurring mistranslation errors [52]. As missense mutations represent more than half of all mutations in the Human Gene Mutation Database (HGMD) and mistranslation-induced misfolding is a potential mechanism for pathologies independent of genomic alterations, we anticipate that understanding the protein quality control pathways that recognize and triage proteins misfolded as the result of missense mutations will gain in importance [53]. A recent study by Sahni *et al*. (2015) found two-thirds of the disease associated missense alleles they tested to have disrupted protein-protein interactions compared to the wild type allele, and approximately 30% of mutant proteins displayed increased binding to components of the protein homeostasis network [54]. While nascent polypeptides that misfold during translation are rapidly degraded, our model substrate has a half life of over four hours at the permissive temperature of 25°C and approximately 90 minutes at 37°C. Our lab has previously reported a panel of temperature sensitive alleles of essential cytosolic genes in *Saccharomyces cerevisiae* and showed that just under half of these alleles have half lives of three hours or less [26]. At the non-permissive temperature of 37°C, Guk1-7’s half life is similar to that of the cytoplasmic model substrate ∆∆ssCPY* (~1 hr) and GFP-Ubc9-2 (~40 min) [15, 22]. Interestingly, by contrast, nuclear temperature sensitive proteins such as Cdc68-1, Sir4-9, Cdc13-1 and Sir3-8, all recognized by the E3 ubiquitin ligase San1, have significantly shorter half lives than the cytoplasmic alleles we have identified [12]. There are a number of potential explanations for why a slower turnover rate is observed. First, the cytoplasmic proteins used in our study and that of Escusa-Toret *et al*. (2013) are constitutively expressed at high levels compared to the low abundance endogenously expressed nuclear proteins examined by Gardner *et al*. (2005)[12, 22]. As a single protein in the cell is represented by a spectrum of folding states, it may be that highly abundant proteins prone to misfolding may only have a fraction of their cellular pool misfolded in such a state as to be recognized and misfolded at one time. Second, temperature sensitive alleles of natural proteins, as opposed to engineered model substrates, have evolved in a cellular context replete with chaperones and protein homeostasis machinery. Given that most chaperones are cytoplasmic, our model substrates have the potential to be recognized and interact with a number of chaperones undergoing refolding cycles before being targeted for degradation. This would be reflected by a slower turnover rate. Finally, we identified the ubiquitin E3 ligase Ubr1 in our screen. Ubr1 alone, or as a double mutant in combination with Gim3 or San1, was not sufficient to completely stop degradation of our model substrate. This would suggest that some misfolded proteins require the activity of a number of E3 ligases for their disposal.

While performing fluorescence microscopy we observed that Guk1-7-GFP forms Q-body like inclusions in the cytoplasm. A similar phenomenon was described for the temperature sensitive Ubc9-2 allele by Escusa-Toret *et al*. (2013)[22]. They speculate that Q-body formation is a rapid early response deployed by the cell to manage misfolded proteins. It would be interesting to see if this inclusion formation can explain the longer half life of our misfolded substrate. A major question is whether proteins sequestered in Q-bodies get redirected to the nucleus for degradation with the help of San1 (as in the case of Pro3-1), or do proteasomes co-localize to Q-bodies as they do with JUNQ inclusions providing a means for substrate disposal in the cytoplasm.

**Fig 11:**
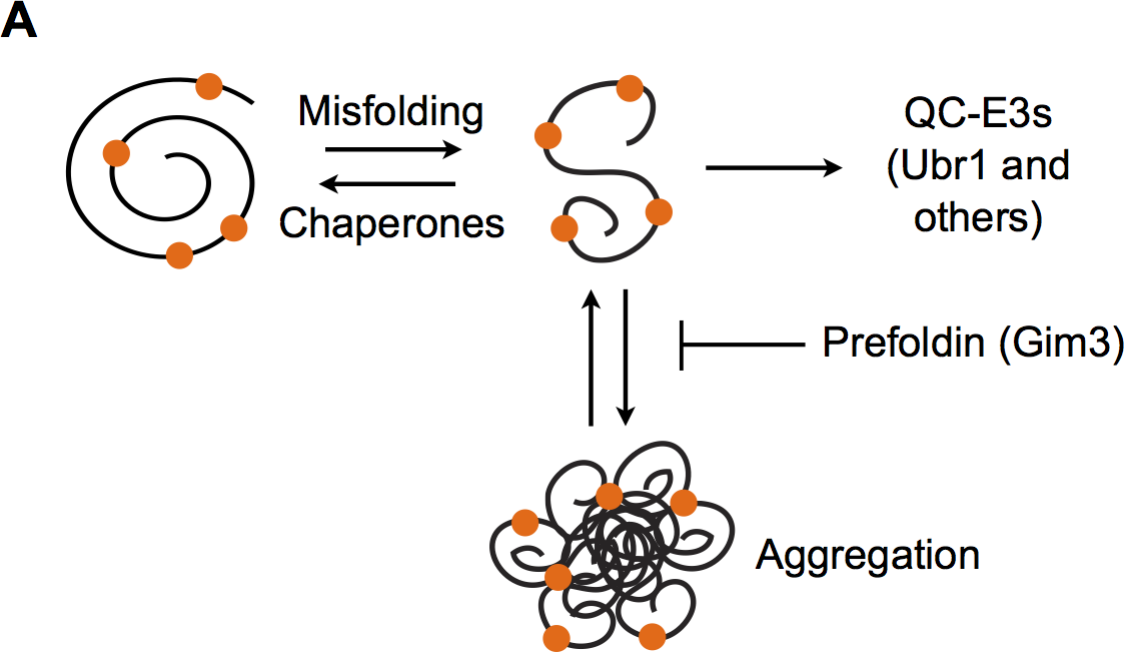
Model for stabilization of temperature sensitive alleles by Gim3. (A) Proposed model for how Gim3 promotes degradation of temperature sensitive alleles destabilized by missense mutations.

In addition to Ubr1, we identified the prefoldin chaperone subunit Gim3 in our screen. We demonstrate that Gim3 was necessary for maintaining Guk1-7 solubility and interacted with our missense allele, but not the wild type protein. In contrast to other reports, we did not find Gim3 to influence ubiquitination of our model substrate, suggesting that it acts independent of substrate ubiquitination (Fig 11). This discrepancy could be explained as being due to previous studies being performed under conditions of proteasome inhibition, or due to the nature of the substrate [55]. Previous reports have demonstrated that knocking down prefoldin subunits results in increased ubiquitination of alpha-synuclein and large inclusion formation, as well as aggregation of huntingtin, an amyloidogenic IPOD substrate [22, 23, 38, 39]. Whereas Abe *et al*. (2013) examined the ubiquitination status of the proteome either in the soluble or pellet fraction under proteasome inhibition, we focused our attention to the soluble fraction of a single substrate[55].

As the structure of the prefoldin complex has no evidence for a nucleotide binding site and therefore lacks ATP-regulated functionality, it is tempting to speculate that prefoldin may act as a holdase [37]. Interestingly, only the mutated and not wild type Guk1 requires Gim3 to remain soluble. In addition, mutations that affect chaperonin components also impaired Guk1-7 degradation. These results indicate that in addition to maintaining misfolded proteins soluble, prefoldin also handed them to the chaperonin for refolding. More work will be required to clearly demonstrate whether Gim3 acts as a holdase to prevent protein aggregation to enhance substrate accessibility for ubiquitin-proteasome mediated degradation. Several other chaperone or co-chaperone proteins have been shown to be important for promoting the degradation of cytosolic misfolded proteins in yeast. Sse1 was shown to help mediate the degradation of the tumor suppressor VHL and is required for the recognition of misfolded proteins by Ubr1 [15, 56]. The Ydj1 J-domain containing Hsp40 mediates both the degradation of ER proteins with exposed misfolded cytosolic domains and the Rsp5 mediated degradation of cytosolic proteins after heat shock [19, 57]. In contrast Sis1, another J-domain containing Hsp40, was shown to be important for the relocalization of cytosolic misfolded proteins to the nucleus [58]. Fes1, an Hsp70 nucleotide exchange factor, was also shown to be important for the degradation of cytosolic misfolded proteins and does so by interacting with the misfolded proteins bound to Hsp70 and triggering their release [59–61]. By demonstrating a role for Gim3 in substrate solubility, our work adds to a growing body of evidence suggesting that prefoldin is important for preventing potentially toxic protein aggregation [39, 62, 63]. In addition to our temperature sensitive alleles, prefoldin has been shown to inhibit human amyloid-beta fibrillation and prevents aggregation of huntingtin [39, 62]. This underscores the potential importance the prefoldin chaperone complex has in maintaining protein homeostasis.

## Materials and Methods

### Yeast strains, plasmids, and media

All yeast deletion strains used in this study are derived from BY4741 or BY4742 wild type (WT) strains and are listed in S1 Table. The temperature sensitive alleles were generously provided by Dr. P. Hieter. The Cup1-Deg1-GFP plasmid was a gift from T. Sommer [41]. The Hsp104-mCherry and Hsp42-mCherry strains were constructed by homologous recombination of a PCR product amplified from a plasmid containing a yeast codon optimized mCherry ORF (BPM 866). Guk1 and Guk1-7 GFP-tagged fusion plasmids (BPM 453, BPM 458) were constructed by inserting ORFs amplified from genomic DNA, with primers containing BamHI and XbaI restriction enzyme recognition sequences, into P_GPD_-GFP(S65T) (BPM 241). Ugp1-3 (BPM 457), Pro3-1 (BPM 507), and Gus1-3 (BPM 500) GFP tagged plasmids were produced in the same fashion using: BamHI and NotI; BamHI and NotI; and NotI and XbaI, respectively. The histidine tagged fusions were produced by cloning PCR amplified inserts into P_GPD_ (BPM 171) using BamHI and SalI (BPM 659, BPM 717). All plasmids used in this study are listed in S2 Table. Cells were grown in synthetic drop out media following standard procedures.

### Stability effect of Guk1-7 mutations

The predicted thermodynamic stability changes of mutations in Guk1-7 were computed using FoldX (version 3.0). The protein structure of Guk1 was downloaded from the Protein Data Bank (PDB accession 1EX7) and was optimized using the repair function of FoldX. Structures corresponding to each of the single point mutations and all four point mutants combined were generated. The predicted effect of mutations on protein structural stability was expressed as the predicted free energy change (∆∆G) and was obtained by subtracting the energy values of the mutant structures from that of the wild type.

### Cellular Thermal Shift Assay (CETSA)

A 50 mL yeast culture grown at 25°C was collected at log phase and harvested by centrifugation. Cells were then lysed with glass beads in 200 μL of native lysis buffer (20 mM HEPES, pH 7.5, 0.5% NP-40, 200 mM NaCl, 1X protease inhibitor mix (Roche), 1 mM 1,10 phenanthroline, 1 mM EDTA). The soluble fraction was collected by centrifugation (16,000 g, 10 min, 4°C) and protein concentration was determined by the DC Protein Assay (BioRad). Samples were normalized to 2 μg/μL and 50 μL aliquots were distributed into PCR strip tubes and run on a PCR machine with the following program: 25°C, 3:00; Gradient 30–50°C, 10:00; 25°C, 1:00. The soluble fraction was once again collected by centrifugation (16,000 g, 10 min, 4°C). Equal volumes were resolved by SDS-PAGE. Membranes were immunoblotted with mouse anti-HIS_6_ (Ablab, 1:2,500) and Cy3 conjugated secondary antibodies (Mandel Scientific, 1:10,000) and quantified using an Odyssey Infrared Imaging System.

### Solubility assay

Yeast cells were grown to log phase at 25°C and then incubated for 20 min at either 25°C or 37°C. Cells were lysed with glass beads in native lysis buffer (20 mM HEPES, pH 7.5, 0.5% NP-40, 200 mM NaCl, 1X protease inhibitor mix, 1 mM 1,10 phenanthroline, 1 mM EDTA) and then precleared by centrifugation at 2,000 g for 5 min at 4°C. Sample protein concentrations were measured by the DC Protein Assay (BioRad) and normalized. Samples were further fractionated into soluble and pellet fractions by centrifugation at 16,000 g for 10 min at 4°C. The pellet fractions were then washed twice with lysis buffer. Equal volumes of total cell lysate, soluble, and pellet fractions were resolved by SDS-PAGE. Samples were analyzed by mouse anti-GFP (Roche, 1:2,500) and rabbit anti-Pgk1 antibodies (Acris Antibodies, 1:10,000) as a loading control.

### Microscopy

Cells were grown in synthetic dropout media lacking histidine to log phase (OD_600_ = 0.8–1.0) at 25°C and then collected at the indicated time points following incubation at 25°C or 37°C with our without 100 μg/mL cycloheximide, as noted. Samples were fixed in 3.7% formaldehyde for 15 minutes at room temperature and then rinsed in 0.1 M potassium phosphate containing 1 M sorbitol before being permeabilized with 0.1% Triton X-100 for ten minutes. Nuclei staining was performed by incubating permeabilized cells in Hoescht 33342 (25 μg/mL) for 10 minutes before mounting cells on slides in mounting media (2% N-Propylgallate, 80% glycerol, 0.02% sodium azide in 1X PBS). Cells were imaged with a Zeiss Axio observer inverted microscope equipped with a 63x oil-immersion objective and a digital camera. Images were analyzed with Zeiss Axiovision software.

### Degradation assay

Cells were grown to log phase in synthetic drop out media at 25°C and cycloheximide was added to a final concentration of 100 μg/mL. Cells were then incubated at either 25°C or 37°C, and at the indicated time points cells were collected by centrifugation. The cells were then resuspended in modified Laemmli buffer (50 mM Tris-HCl, pH 6.8, 2% SDS, 10% glycerol), and lysed with glass beads. Protein concentration was assessed by the DC Protein Assay (BioRad). Equal amounts of protein were resolved by SDS-PAGE following the addition of 10X 2-mercaptoethanol (20%) and dye to each sample. Immunoblots were performed with a mouse anti-GFP primary antibody (Millipore, 1:2,500) and a rabbit anti-Pgk1 (1:10,000, Acris Antibodies) as a loading control. Infrared secondary antibodies were used (Mandel Scientific, 1:10,000) and membranes were scanned and analyzed with an Odyssey Infrared imaging system (LI-COR).

### Flow cytometry

Yeast cells were grown in synthetic drop out media to log phase before the addition of 100 μg/mL cycloheximide and incubated at 25°C or 37°C as indicated. Samples were run on a BD FACSCalibur instrument (BD Biosciences) with a 488 laser and GPF was detected with a 530/30 filter. 50,000 events were collected. Analysis was performed with FlowJo (FlowJo Data Analysis Software, LLC). For multi-hour CHX chase experiments median GFP fluorescence values were normalized to that of the first time point. FACS sorting was performed with a BD Influx instrument by the UBC Flow facility.

### GFP pulldown

Gim3-TAP yeast cells transformed with a control empty vector (BPM 42), P_GPD_-GFP (BPM 779), P_GPD_-Guk1-GFP (BPM 780), or P_GPD_-Guk1-7-GFP (BPM 781) were grown to log phase and then lysed with glass beads and native lysis buffer (20 mM HEPES, pH 7.5, 0.5% NP-40, 200 mM NaCl, 1X protease inhibitor mix, 1 mM 1,10 phenanthroline, 1 mM EDTA, 10 mM iodoacetamide). To pulldown GFP-tagged proteins, lysates were incubated for 2 hours at 4°C with 20 μL GFP-Trap coupled agarose beads (Chromotek). Beads were washed three times in lysis buffer before samples were eluted with 3X SDS sample buffer. Nitrocellulose membranes were probed with mouse anti-GFP (Roche, 1:2,500), rabbit anti-Pgk1 (Acris Antibodies, 1:10,000), rabbit anti-TAP (Fisher, 1:2,500), and mouse anti-ubiquitin (Millipore, 1:2,500) primary antibodies.

### Proteasome function

Yeast cultures were grown to saturation in synthetic drop out media overnight at 30°C and then diluted to OD_600_ = 0.2 and left to grow for 3 hours at 30°C. 100 μM copper sulphate was added to the culture and incubated at 30°C for 4 hours. An initial sample was removed and then cycloheximide was added to the culture to a final concentration of 100 μg/mL. Samples were collected at the indicated time points. Cells were lysed with glass beads and lysis buffer (1% Tx-100, 0.1% SDS, 150 mM NaCl, 5 mM EDTA, 50 mM Tris-HCl, pH 7.5, 1 mM PMSF, 1X protease inhibitor mix). Protein concentrations were assessed using the DC Protein Assay (BioRad) and equal amounts were resolved by SDS-PAGE.

### Statistical analysis

Unpaired two tailed Student’s t-tests were used to assess significance of differences between wild type and *gim3∆* or *ubr1∆* strains. One-way ANOVA with post-hoc Tukey HSD (honest significant difference) was used to assess significance of differences between multiple deletion strains.

## Supporting Information

S1 Fig Guk1-7-GFP flow cytometry. (A) Box plot of quantification for fluorescence microscopy images in Fig 2A. Corrected total cell fluorescence was calculated by subtracting the mean fluorescence of background readings from the integrated density. n = 108, 120, 101, and 168 for Guk1 25°C, Guk1 37°C, Guk1-7 25°C, and Guk1-7 37°C, respectively. (B) Wild type cells expressing Guk1-GFP or Guk1-7-GFP on a plasmid and expressed from their endogenous promoters were incubated at 25°C or 37°C with CHX. Samples were collected at the indicated time points and analyzed by flow cytometry. (C) Flow cytometry validation experiments for the deletion strains identified by barcode sequencing. Cells expressing Guk1-7-GFP were incubated with CHX at 25°C or 37°C for two hours prior to flow cytometry analysis. Note that expression of *YOR364W* and *RIM15* from a plasmid (*i*.*e*., add back experiments) failed to rescue the phenotype indicating that an additional mutation may have caused stabilization of the model substrate. Deletions of *UBR1* and *GIM3* were further analyzed in this work but not *MUP3* and *BRA7*.
(TIFF)

S2 Fig Ubr1 does not act with San1 in the degradation of Guk1-7-GFP. (A) Guk1-GFP was expressed in wild type or *ubr1∆* cells and incubated with cycloheximide for 2 hours at 25°C or 37°C prior to performing flow cytometry. The results represent the relative fluorescence intensities and standard deviations from three independent experiments. Statistical significance was tested using an unpaired two tailed Student’s t-test. (B) Guk1-7-GFP was expressed in wild type, *ubr1∆*, *san1∆*, and *ubr1∆san1∆* cells and incubated with cycloheximide for 2 hours at 25°C or 37°C prior to performing flow cytometry. The results represent the average and standard deviations from three independent experiments. Statistical significance was tested using a one-way ANOVA and a Tukey HSD post-hoc test. *, **, and ns denote P < 0.05, P < 0.01, and not significant, respectively. (C) Pro3-1-GFP expressing cells were grown and treated as in B. Samples were analysed using a one-way ANOVA followed by Tukey’s post hoc test, ** denotes P <0.01.
(TIFF)

S3 Fig Guk1-7-GFP Gim3 interaction and viability assay. (A) Guk1-7-GFP was immunoprecipitated from wild type or Gim3-TAP expressing cells incubated at 25°C and then immunoblotted with anti-TAP, anti-GFP, or anti-Pgk1 antibodies. (B) Viability assay. Wild type, *gim3∆*, *guk1-7*, or double *guk1-7*, *gim3∆* cells were streaked on rich media plates and incubated for two days at the indicated temperatures.
(TIFF)

S1 Table Yeast strains used in this study. (XLSX)

S2 Table Plasmids used in this study. (XLSX)
