# Supplementary figures and images for: Prefoldin Promotes Proteasomal Degradation of Cytosolic Proteins with Missense Mutations by Maintaining Substrate Solubility
Source: PLoS Genet. 2016 Jul 22;12(7):e1006184. doi: 10.1371/journal.pgen.1006184 (PMC4957761; doi:10.1371/journal.pgen.1006184)

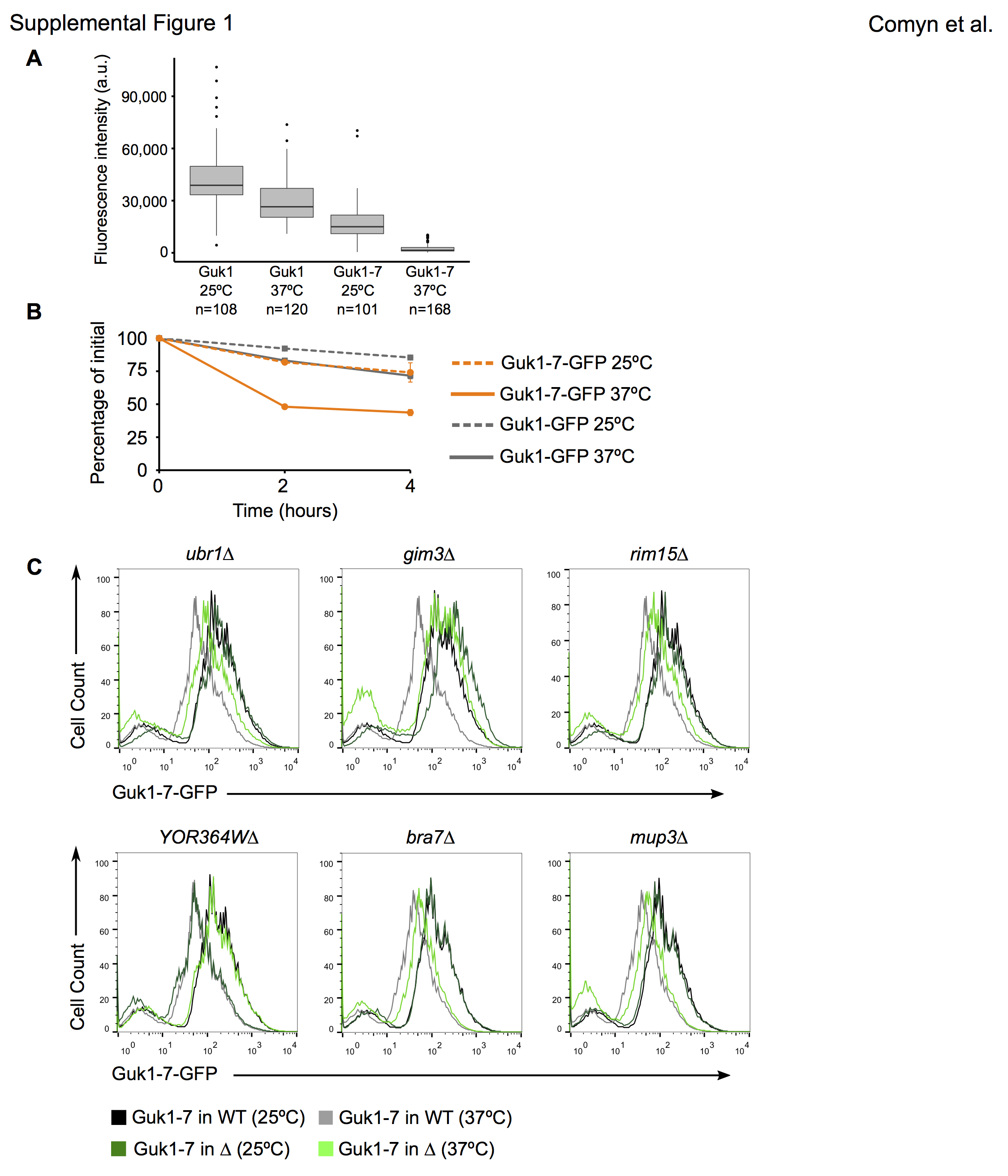

Supplement: S1 Fig — (A) Box plot of quantification for fluorescence microscopy images in Fig 2A. Corrected total cell fluorescence was calculated by subtracting the mean fluorescence of background readings from the integrated density. n = 108, 120, 101, and 168 for Guk1 25°C, Guk1 37°C, Guk1-7 25°C, and Guk1-7 37°C, respectively. (B) Wild type cells expressing Guk1-GFP or Guk1-7-GFP on a plasmid and expressed from their endogenous promoters were incubated at 25°C or 37°C with CHX. Samples were collected at the indicated time points and analyzed by flow cytometry. (C) Flow cytometry validation experiments for the deletion strains identified by barcode sequencing. Cells expressing Guk1-7-GFP were incubated with CHX at 25°C or 37°C for two hours prior to flow cytometry analysis. Note that expression of YOR364W and RIM15 from a plasmid (i.e., add back experiments) failed to rescue the phenotype indicating that an additional mutation may have caused stabilization of the model substrate. Deletions of UBR1 and GIM3 were further analyzed in this work but not MUP3 and BRA7. (TIFF) [file pgen.1006184.s001.tiff]

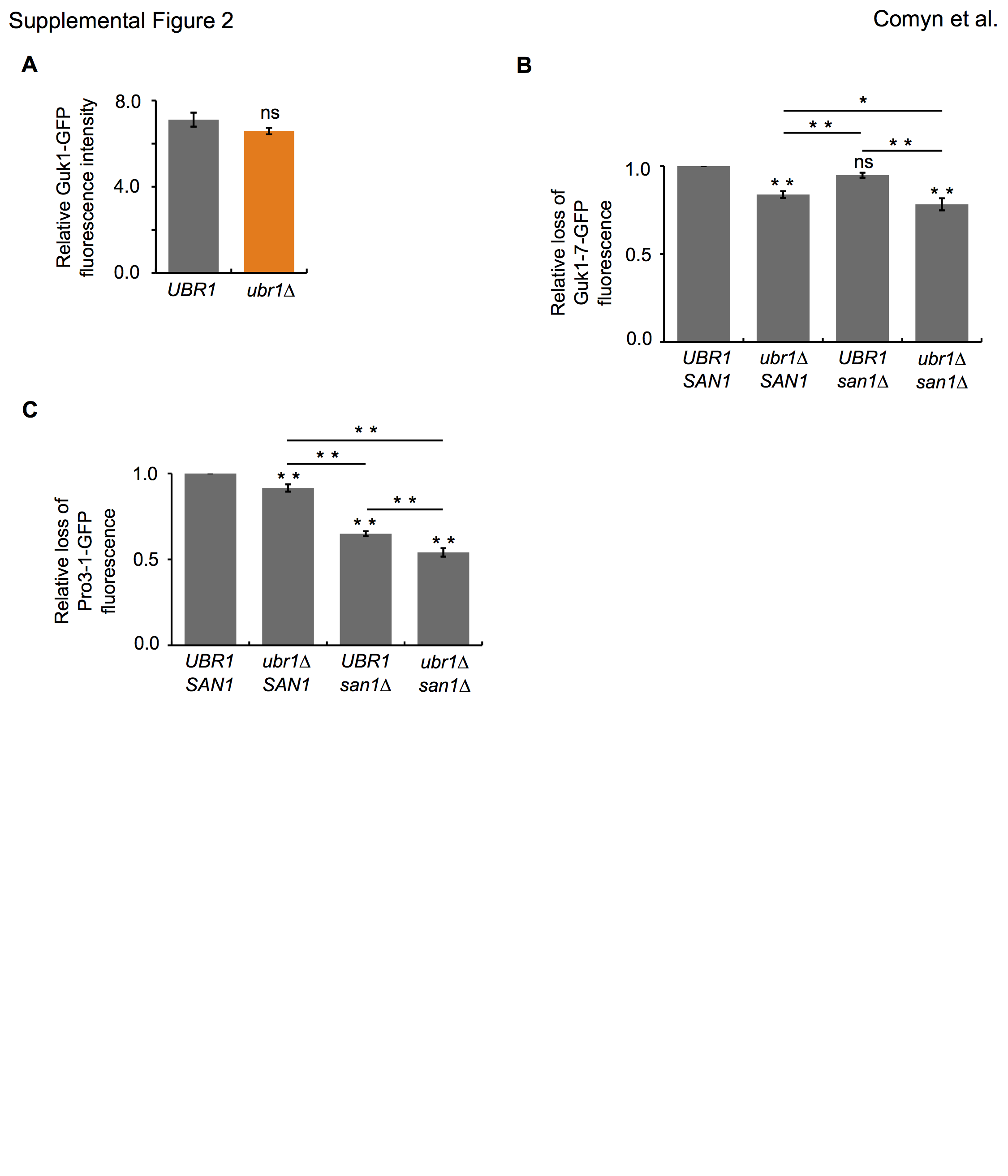

Supplement: S2 Fig — (A) Guk1-GFP was expressed in wild type or ubr1∆ cells and incubated with cycloheximide for 2 hours at 25°C or 37°C prior to performing flow cytometry. The results represent the relative fluorescence intensities and standard deviations from three independent experiments. Statistical significance was tested using an unpaired two tailed Student’s t-test. (B) Guk1-7-GFP was expressed in wild type, ubr1∆, san1∆, and ubr1∆san1∆ cells and incubated with cycloheximide for 2 hours at 25°C or 37°C prior to performing flow cytometry. The results represent the average and standard deviations from three independent experiments. Statistical significance was tested using a one-way ANOVA and a Tukey HSD post-hoc test. *, **, and ns denote P < 0.05, P < 0.01, and not significant, respectively. (C) Pro3-1-GFP expressing cells were grown and treated as in B. Samples were analysed using a one-way ANOVA followed by Tukey’s post hoc test, ** denotes P <0.01. (TIFF) [file pgen.1006184.s002.tiff]

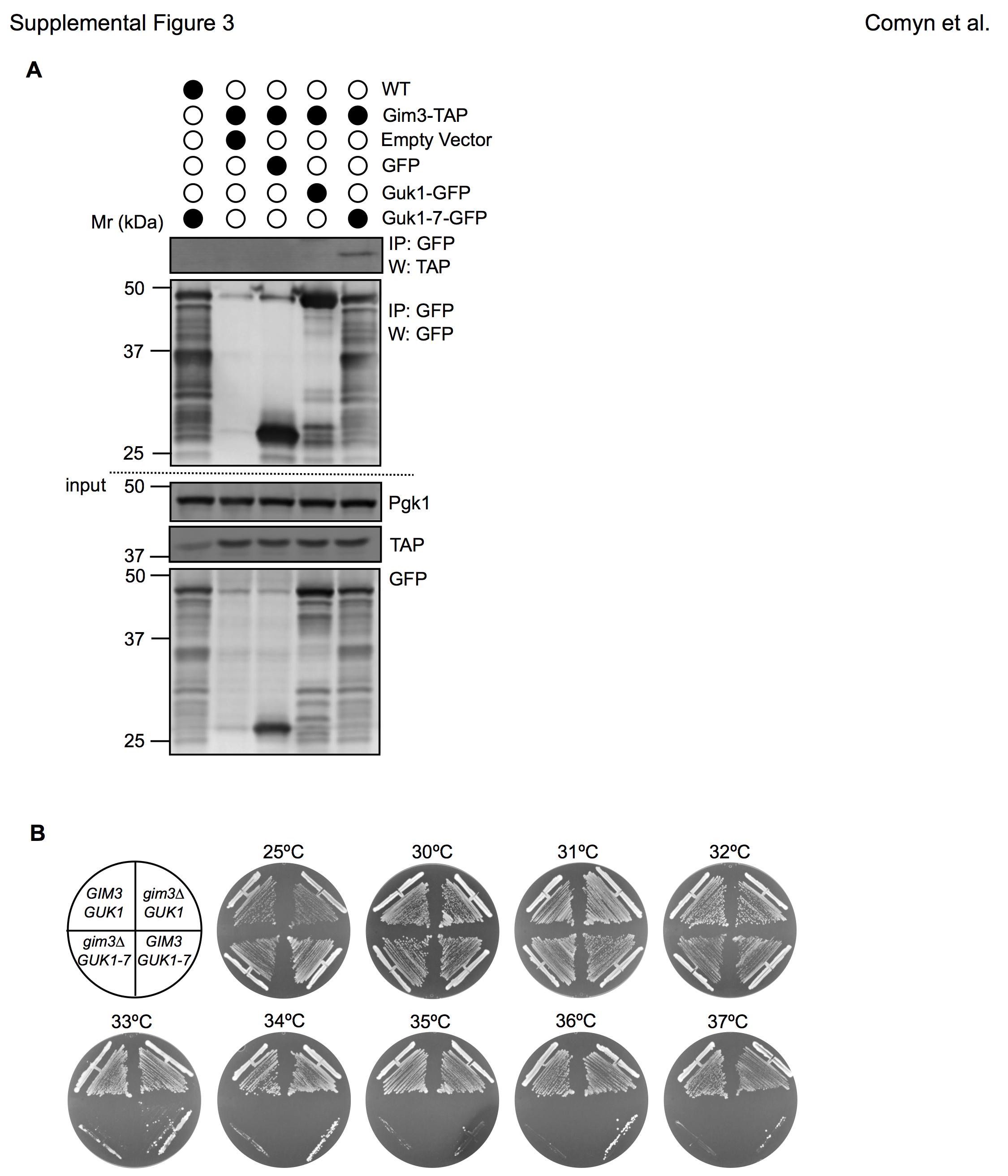

Supplement: S3 Fig — (A) Guk1-7-GFP was immunoprecipitated from wild type or Gim3-TAP expressing cells incubated at 25°C and then immunoblotted with anti-TAP, anti-GFP, or anti-Pgk1 antibodies. (B) Viability assay. Wild type, gim3∆, guk1-7, or double guk1-7, gim3∆ cells were streaked on rich media plates and incubated for two days at the indicated temperatures. (TIFF) [file pgen.1006184.s003.tiff]
